# Functional characterization of Gh_A08G1120 (GH3.5) gene reveal their significant role in enhancing drought and salt stress tolerance in cotton

**DOI:** 10.1186/s12863-019-0756-6

**Published:** 2019-07-23

**Authors:** Joy Nyangasi Kirungu, Richard Odongo Magwanga, Pu Lu, Xiaoyan Cai, Zhongli Zhou, Xingxing Wang, Renhai Peng, Kunbo Wang, Fang Liu

**Affiliations:** 1 State Key Laboratory of Cotton Biology/Institute of Cotton Research, Chinese Academy of 15 Agricultural Sciences (ICR, CAAS), Anyang, 455000 Henan China; 2 School of Biological and Physical Sciences (SBPS), Jaramogi Oginga Odinga University of Science and Technology (JOOUST), Main Campus, 210-40601, Bondo, Kenya; 3 Research Base in Anyang Institute of Technology, State Key Laboratory of Cotton Biology/ Anyang Institute of technology, Anyang, 455000 Henan China

**Keywords:** Cis-regulatory elements, Virus induced gene silencing, Gretchen Hagen3, Orthologous genes

## Abstract

**Background:**

Auxins play an important role in plant growth and development; the auxins responsive gene; auxin/indole-3-acetic acid (Aux/IAA), small auxin-up RNAs (SAUR) and Gretchen Hagen3 (GH3) control their mechanisms. The *GH3* genes function in homeostasis by the catalytic activities in auxin conjugation and bounding free indole-3-acetic acid (IAA) to amino acids.

**Results:**

In our study, we identified the *GH3* genes in three cotton species; *Gossypium hirsutum, Gossypium arboreum* and *Gossypium raimondii,* analyzed their chromosomal distribution, phylogenetic relationships, cis-regulatory element function and performed virus induced gene silencing of the novel *Gh_A08G1120 (GH3.5)* gene*.* The phylogenetic tree showed four clusters of genes with clade 1, 3 and 4 having mainly members of the GH3 of the cotton species while clade 2 was mainly members belonging to Arabidopsis. There were no paralogous genes, and few orthologous genes were observed between *Gossypium* and other species. All the GO terms were detected, but only 14 genes were found to have described GO terms in upland cotton, more biological functions were detected, as compared to the other functions. The GH3.17 subfamily harbored the highest number of the cis-regulatory elements, most having promoters towards dehydration-responsiveness. The RNA expression analysis revealed that 10 and 8 genes in drought and salinity stress conditions respectively were upregulated in *G. hirsutum*. All the genes that were upregulated in plants under salt stress conditions were also upregulated in drought stress; moreover, *Gh_A08G1120 (GH3.5)* exhibited a significant upregulation across the two stress factors. Functional characterization of *Gh_A08G1120 (GH3.5)* through virus-induced gene silencing (VIGS) revealed that the VIGS plants ability to tolerate drought and salt stresses was significantly reduced compared to the wild types. The chlorophyll content, relative leaf water content (RLWC), and superoxide dismutase (SOD) concentration level were reduced significantly while malondialdehyde concentration and ion leakage as a measure of cell membrane stability (CMS) increased in VIGS plants under drought and salt stress conditions.

**Conclusion:**

This study revealed the significance of the *GH3* genes in enabling the plant’s adaptation to drought and salt stress conditions as evidenced by the VIGS results and RT-qPCR analysis.

**Electronic supplementary material:**

The online version of this article (10.1186/s12863-019-0756-6) contains supplementary material, which is available to authorized users.

## Background

The *Gossypium* species are important cash crops and the cornerstone for the survival of the textile industries globally being the chief source of raw materials [1]. Globally cotton fiber is known as the white gold, and its production is key to the economies of several countries worldwide [2]. Despite the economic importance of cotton, its production has undergone a series of challenges and a decline due to the combined effects of abiotic and biotic stress factors [3]. A number of advancements have been made in order to develop a more stress tolerance cotton, for instance; the development of BT-cotton has significantly improved cotton production globally [4]. In relation to abiotic stress factors such as drought, salt and extreme temperatures have continued to pose a challenge in cotton production, since they are region specific and purely regulated by the climatic conditions and human activities. It is estimated that over 6% of the agronomic lands are saline, and losses due to drought stress are estimated to be at 30% globally [5]. Improvement of cotton through the conventional method has resulted in limit success, due to the narrow genetic base of the cultivated cotton and genetic male sterility [6, 7]. Adoption of molecular technology is the most appropriate method to develop more resilient and highly adaptive cotton genotypes to various abiotic stress factors. Several stress responsive genes have been investigated in cotton and found to be effective in improving their adaptability, for instance, the late embryogenesis abundant (LEA) proteins [8], the *NAC* gene the cyclin dependent kinase (CDK) gene [9], G-protein-coupled receptors (GPCRs) gene [10], the multidrug and toxic compound extrusion (*MATE*) gene and the *MYB* genes [11], among others.

The auxins play an important role in plant growth and development; they are produced by both plants and plant pathogens in the regulation of plant growth [12]. Most of the regulatory mechanisms of auxin are controlled by auxin-responsive genes; these genes are classified into three groups they include Aux/IAA, SAUR and GH3 [13]. The auxins have been associated with tropic responses by controlling cell division and elongation [14], biotic and abiotic stress factors [15]. Insufficient amounts of auxins could lead to an abnormal reduction of growth in plants; the first group is the IAA, which is the main auxin as signaling molecules and promote cell division and cell elongation in the shoot and roots [16]. The second group is the SAUR although their roles have not clearly been identified, they have been linked with the regulation of a wide range of physiological, cellular and developmental processes [17].

The last group of auxins is the GH3 protein family, which are mainly involved in homeostasis by the catalytic activities in auxin conjugation and bounding free IAA to amino acids [18]. However few studies have been done on these genes in relation to environmental stresses, for instance, it has been found that the negative feedback regulation of the *GH3* genes in transformed Arabidopsis seedlings, suppressed the auxin response to salicylic acid, salt, abscisic acid (ABA) and cold stress treatments [19]. The GH3 family members were first isolated in soybean hypocotyls [20], and their functions have been studied in various plants, in the studies conducted in Arabidopsis the GH3 have been categorised into three main groups based on their substrate specificity and sequence similarities [21]. The group I GH3 enzymes are jasmonic acids (JA) -amido or salicylic acid (SA) -amido synthetases, Group II GH3 functions in the negative feedback regulation of IAA concentration by conjugation of IAA to the free IAA and store phytohormone while group III, are involved in salicylic acid signalling [22].

The *GH3* gene studies have been reported in several plants; *DFL1*, an auxin-responsive *GH3* gene was reported to promote light response of hypocotyl and reduce shoot cell elongation and lateral root formation in Arabidopsis [23], In *Malus sieversii GH3* genes were significantly induced after various phytohormones and abiotic stress treatments, [19], in *Medicago truncatula GH3* genes were found to mediate IAA homeostasis in the regulation of nodule formation [24], and in moss plant, the GH3 proteins play a pertinent role in auxin homeostasis by conjugating excess of active free auxin to inactive IAA-amide conjugates [25]. In *Oryza sativa* the *GH3* genes are associated with suppression of pathogen-induced IAA accumulation by down-regulating auxins signalling [26]. Currently, no studies have been conducted on the relationship between the *GH3* genes and abiotic stresses in cotton plants. The complete sequencing of the three representative genomes in *G. hirsutum* [27], *G. raimondii* [28] and that of *G. arboreum* [29] has provided a platform for transcription analysis of the various cotton genes*.* In this study, we carried out genome-wide identification of GH3 proteins in the three cotton species, *G. hirsutum, G. arboreum* and finally in *G. raimondii.* We further analyzed the functional characterization of the *Gh_A08G1120 (GH3.5)* the novel gene in *G. hirsutum*. The functional characterization of this gene was done through gene knockdown mechanism, and the VIGSthe virus induced gene silenced (VIGS) cotton seedlings evaluated under drought and salt stresses.

## Methods

### Identification of proteins encoded by the *GH3* genes in the cotton genome

The annotations for The *G. arboreum* of A genome GH3 protein sequences were downloaded from the Beijing Genome Institute database (https://www.bgi.com/) *G. hirsutum* belonging to the AD genome GH3 protein sequences were downloaded from the Cotton Research Institute website (http://mascotton.njau.edu.cn), while those of *G. raimondii* of the D genome was obtained from Phytozome (http://www.phytozome.net/). The conserved domain of GH3 proteins (PF03321) was downloaded from Pfam protein families (http://pfam.xfam. org). The GH3 proteins were then queried using the Hidden Markov Model (HMM) searches of the sequences in the downloaded FASTA file using the HMMER [30] against *G. hirsutum*, *G. raimondii* and *G. arboreum* protein sequences. The amino acids sequences were analyzed for the presence of the GH3 protein domains by ScanProsite tool (http://prosite.expasy.org/scanprosite/) and SMART program (http://smart.embl -heidelberg .de/).

### Construction of phylogenetic tree and subcellular localization analyses of the proteins encoded by the *GH3* genes

The GH3 protein sequences from Arabidopsis (http://www.arabidopsis.org/), rice (http://rice.plantbiology. msu.edu/index.shtml) together with the three cotton species; *G. hirsutum*, *G. arboreum*, and *G. raimondii* were aligned by “Muscle”, using the neighbor-joining method in MEGA 6.06 and a maximum likelihood tree was generated [31] with a bootstrap value of 1000 were used to investigate the evolutionary history of these genes. A phylogenetic tree was constructed, the multiple sequence alignments of all the GH3 proteins were performed by Clustal omega, MEGA 7.0 software using default parameters [32]. The physiochemical characteristics of all the obtained GH3 proteins were determined through an online ExPASy Server tool (http://www.web.xpasy.org/ compute_pi/). In addition, subcellular location prediction for all the upland cotton GH3 proteins was determined through Wolfpsort (https://www.wolfpsort.hgc.jp/). The subcellular prediction results were confirmed using other two online tools TargetP1.1 server [33] and Protein Prowler Subcellular Localization Predictor version 1.2 (http://www.bioinf.scmb. uq.edu.au/pprowler_webapp_ 1–2/).

### Gene ontology (GO) annotation, RNA-seq data analysis, and Cis-regulatory elements analysis

The *GH3* genes were analysed using Blast2GO software (https://www.blast2go.com/) for the gene ontology (GO) terms. Genes were analyzed for three categories of GO classification: molecular function (MF), biological processes (BP), and cellular components (CC). The expression patterns of the upland cotton (*G. hirsutum*) *GH3* genes were analyzed using RNA-seq data from various tissue/stages of development obtained from the cotton functional genome database (https://cottonfgd.org/). The selected raw data were transformed by log 2, and then HemI software [34] was used to visualize the expression. The promoter sequences of all the GH3 genes were obtained from the cotton genome project. Transcriptional response elements of the *GH3* gene cis-regulatory elements were predicted using the Plant care server, a database of plant cis- regulatory elements and the Insilco analysis of the promoter sequences (http://bioinformatics.psb. ugent.be/ web tools/plant care/html/).

### Plant material and treatment for analysis of the expression levels of the cotton *GH3.5* genes under drought and salt stress conditions

To evaluate the stress response of *GH3.5* genes in cotton, *G. hirsutum* an upland tetraploid cotton mainly grown for its high production, but highly susceptible to various abiotic stress condition was used. An accession number CRI-12, a species of upland tetraploid cotton, was chosen for this experiment, the cotton germplasm is mainly grown in China due to its high productivity ability, though relatively less tolerant to various abiotic stresses. The seeds were obtained from the Cotton Research Institute of Chinese Academy of Agricultural Sciences, China. The cotton seeds were germinated on wet filter paper for 3 days at 25 °C in a growth chamber. The seedlings were then transferred to well conditioned room, and were grown in a Hoagland nutrient solution [35], under hydroponic setup. The greenhouse conditions set at 28 day/25 night, 14 h photoperiod, and 60–70% relative humidity. At three leaf stage, the seedlings were subjected to stress, by transferring to a nutrient solution with 250 mM sodium chloride (NaCl) and 15% PEG-6000, for salt and drought stress treatment, respectively. Root, stem and leaf tissues were harvested at 0 h, 1 h, 3 h, 6 h, and 12 h of posttreatment. Untreated plants served as the control. The samples were then immediately frozen in liquid nitrogen on collection, and stored at − 80 °C awaiting RNA extraction.

### RNA extraction and quantitative RT-qPCR analysis

Total RNA was extracted using EASYspin plus plant RNA kit (Aidlab, Biotech, Beijing, China), following the manufacturer’s instructions. The RNA quality and quantity were determined using Nanodrop 1000 spectrophotometer RNA samples with 260/280 ratio between 1.8 and 2.1 and 260/230 ratio between 2.0 and 2.5 were used for cDNA synthesis. Primers were designed for all genes using Primer Premier 5 software (Additional file 1: Table S1). The cotton *GhActin* gene forward sequence 5’ATCCTCCGTCTTGACCTTG3’ and reverse sequence 5’TGTCCGTCAGGCAACTCAT3’ was used as the reference standardization for the RT-qPCR analysis. We conducted a BLAST search to identify the specificity of each pair of primers. Fast Start Universal SYBRgreen Master (Rox) (Roche, Mannheim, Germany) was used to perform RT-qPCR in accordance with the manufacturer’s instructions. Reactions were prepared in a total volume of 20 ml, comprising 10 ml of SYBR green master mix, 2 ml of cDNA template, 6 ml of ddH_2_O, and 2 ml of each primer for a final concentration of 10 mM. The PCR thermal cycling conditions were as follows: 95 °C for 10 min, 40 °C cycles of 95 °C for 5 s, 60 for 30 s, and 72 °C for 30 s. Data were collected during the extension step: 95 °C for 15 s, 60 °C for 1 min, 95 °C for 30 s, and 60 °C for 15 s. For each tissue, at least three independent biological replicates and three technical replicates of each biological replicate were taken for the analysis.

### Functional characterization of the *Gh_A08G1120 (GH3.5)* novel gene through virus induced gene silencing (VIGS)

RNAi technique was applied using tobacco rattle virus (TRV) [36]. Fragments from the coding DNA sequence with 1719 bp of *Gh_A08G1120 (GH3.5)* was amplified from *G. hirsutum* cv. TM-1 cDNA, and subsequently introduced into the TRV: 00 plasmid. The DNA was digested with restriction enzymes *sac1* and *xhoI* to generate the TRV: *Gh_A08G1120 (GH3.5).* The TRV: *Gh_A08G1120 (GH3.5)* and TRV1 construct were inserted into *A. tumefaciens* strain GV3101 by electroporation. We followed the same procedure for VIGS in cotton as described by Li et al. [34]. The novel gene fragments were amplified from the upland cotton *G. hirsutum* cDNA using PCR, and a pTRV2-*Gh_A08G1120 (GH3.5)* constructs were generated by inserting *SacI* 5’C**GAGCTC**GGAAAACCAATCACCACCA3’ and *XhoI* 5’C**CTCGAG**GGAAAATCAGCC CACACAA3’ digested PCR fragments of *Gh_A08G1120* into the pTRV2 vector. Cultures of the LBA4404 *Agrobacterium* strain containing the pTRV1, pTRV2, pTRV2-PDS and the pTRV2-*Gh_A08G1120*-vectors, were cultured at 28 °C in Luria−Bertani (LB) liquid medium (pH 5.6) with 10 mM 2-(N-morpholino)-ethanesulfonic acid (MES). A volume of 20 μM acetosyringone, kanamycin, and rifampicin antibiotics added before put in the incubating shaker at 180 rpm (rpm), for 12 h, at the end the OD was determined at 1.5. After 12 h of shaking, the cells were centrifuged at 8000 rpm for 10 min and re-suspended in infiltration buffer containing 10 mM of magnesium chloride (MgCl2), 10 mM MES of pH 5.6, and 200 μM acetosyringone to a final OD_600_ = 1.5, the mixture was left for 3 h at room temperature. The re-suspension of pTRV1 was mixed with pTRV2, pTRV2-PDS and pTRV2-*Gh_A08G1120 (GH3.5)*, separately, at a ratio of 1:1, after which the infusion was ready for infiltration. The approximately 3 ml of the infiltration medium containing the *Agrobacterium* strain was infiltrated into the cotton cotyledons before the emergence of the first true leaf. The seedling infiltrated with pTRV1 and pTRV2, were used as negative controls. Each inoculation was carried out three times, and six seedlings were infiltrated for each construct. When the VIGS phenotype became visible, the leaf, root and stem samples were collected, and stored at − 80 °C and RNA extracted in order to determine the expression levels of the two silenced genes in the three organs. The genes specific primer sequence for *Gh_A08G1120* was designed, the forward sequence 5’CAATGAAAGC AATGCAGTCA3’ and reverse sequence 5’AAATCAGCCCACACAAGAGA3’ primers were designed for the RT-qPCR analysis. At the three leaf stage, the plants were subjected to drought and salt stress treatment, and samples collected for further analysis at 0 h, 3 h and 24 h post tress treatment.

### Physiological and biochemical evaluations of the VIGS and non-VIGS cotton seedings under drought and salt stress conditions

The VIGSVIGS and the wild type cotton seedlings were evaluated under drought and salt stress conditions at three true leaf stages after infusion. The chlorophyll content, relative leaf water content (RLWC), and cell membrane stability (CMS) were measured. The chlorophyll content, CMS through ion leakage and RLWC were determined as described by Magwanga et al. [37]. Furthermore, two antioxidant and a single oxidant enzyme were evaluated among the VIGS-plants and wild type, proline levels, Superoxide dismutase (SOD) and malondialdehyde (MDA) were measured as previously described by Magwanga et al. [38]. The traits measured have been applied extensively in evaluating various field crops for water stress tolerance [39].

### Stress responsive genes profiling on the VIGS-plants and non-VIGS cotton plants under drought and salt stress conditions

We applied three known stress responsive genes in evaluating their expression levels in both VIGS and none VIGS cotton seedlings exposed to drought and salt stress conditions. The genes used were *GhP5CS, GhMYB* and *GhSOD* (Table 1). A superoxide dismutase gene (*TaSOD2)* has been found to be responsible for enhancing salt stress tolerance in wheat [40]. Moreover, expression of *SOD* and ascorbate peroxidase (APX) genes do promote growth and yield of Arabidopsis under salt stress [41]. Furthermore, the MYB transcription factor family play a central role in triggering the right responses in enhancing abiotic stress tolerance in plants [42].

**Table 1:** Stress responsive genes

GENE name	Gene ID	forward	reverse
Pyrroline-5-carboxylate synthase	(*P5CS*)	TTGAAATAGTGGACGACGTGGC	CTCAGCGCCTAGACCAAATCG
Superoxide dismutase	(*SOD*)	CATCTCTCACGCACTCTGTC	CCTTAGCCATTTCTGTCTGTG
Myeloblastosis	(*MYB*)	TGGGAGTAGAGGAGGAGAAGC	TTGAGGTGCCTGTGGATTG

## Results

### Identification and sequence analysis of the GH3 proteins in cotton

The availability of the whole sequences for the three cotton species enabled us to identify the GH3 proteins harbored in their genome. The Pfam domain PF03321 was used as the query to obtain the GH3 proteins, 58, 38 and 36 GH3 proteins were identified in *G. hirsutum*, *G. raimondii* and *G. arboreum*, respectively, but after validating all the GH3 sequences through ScanProsite tool (http://prosite.expasy.org/scanprosite/) and simple modular architecture research tool (SMART) scan program (http://smart.embl -heidelberg .de/) as described by Yuan et al. [19] in the identification of the GH3 proteins in *Malus sieversii* Roem. The number of the GH3 proteins obtained from the three cotton species is in agreement with previous reports, in which relatively few numbers of the GH3 proteins have been identified in various dicotyledonous plants such as chickpea, soybean, Medicago and Lotus, with 11, 28, 10, and 18 *GH3* gene members, respectively [24]. Similarly, the apple plant had 29 candidates of the GH3 family proteins in its genome [19]. Moreover, the proportions of the proteins encoded by the *GH3* genes in the three cotton species, 58 (AD), 38 (D) and 36 (A), showed that there was an element of gene loss in either during the emergence of tetraploid cotton (AD), being the number of the proteins encoded by the *GH3* genes were less than the sum total of either of the two diploid cotton species. The AD genome emerged due to whole genome duplication between A and D [43]. The gene loss phenomenon has been observed in a number of stress responsive genes, such as the *LEA* [8], thus the loss could be attributed to either chromosome rearrangement or shortening. In the analysis of the various physiochemical properties of the GH3 proteins, their protein properties were varied. For the GH3 proteins obtained from the tetraploid cotton, their protein lengths ranged from 114 aa (*Gh_A01G0776*_ *GH3.12*) to 1137 aa (*Gh_D08G0262*_ *GH3.17*); molecular weights ranged from 13.01 kDa to 126.749 kDa; isoelectric value ranged from 4.997 to 10.162 and their grand hydropathy values (GRAVY) ranged from − 0.432 to − 0.051. In *G. arboreum* the protein length ranged from 461 aa to 1116 aa, molecular weights ranged from 51.708 kDa to 124.403 kDa, the pI ranged from 4.987 to 8.727 while their GRAVY values ranged from − 0.322 to − 0.039. Similar ranges were also observed among the GH3 proteins for *G. raimondii*, for instance, the protein lengths ranged from 68 aa to 1137 aa, molecular weight ranged from 8.024 kDa to 126.531 kDa and their GRAVY values ranged from − 0.478 to − 0.051. It is interesting to note that all the GH3 proteins obtained from the three cotton species, had negative GRAVY values, and ranged from − 0.478 to − 0.039 an indication that the proteins encoded by the *GH3* genes were hydrophilic in nature (Additional file 2: Table S2). Hydrophilicity is associated with a number of proteins responsible for enhancing desiccation tolerance. Chakrabortee et al. [44] observed that the adaptation of desiccation-tolerant organisms is due to the widespread abundance of hydrophilic proteins, such as the LEA proteins. A similar observation was made by Magwanga et al. [8] in the analysis of the LEA proteins in cotton under drought stress conditions, thus the high hydrophilicity index among the GH3 proteins provided a stronger indication that these proteins are playing an integral role in enhancing drought stress tolerance in cotton.

### Chromosomal mapping, subcellular localization prediction and Cis-regulatory element analysis of the cotton GH3 proteins

**Fig. 1:**
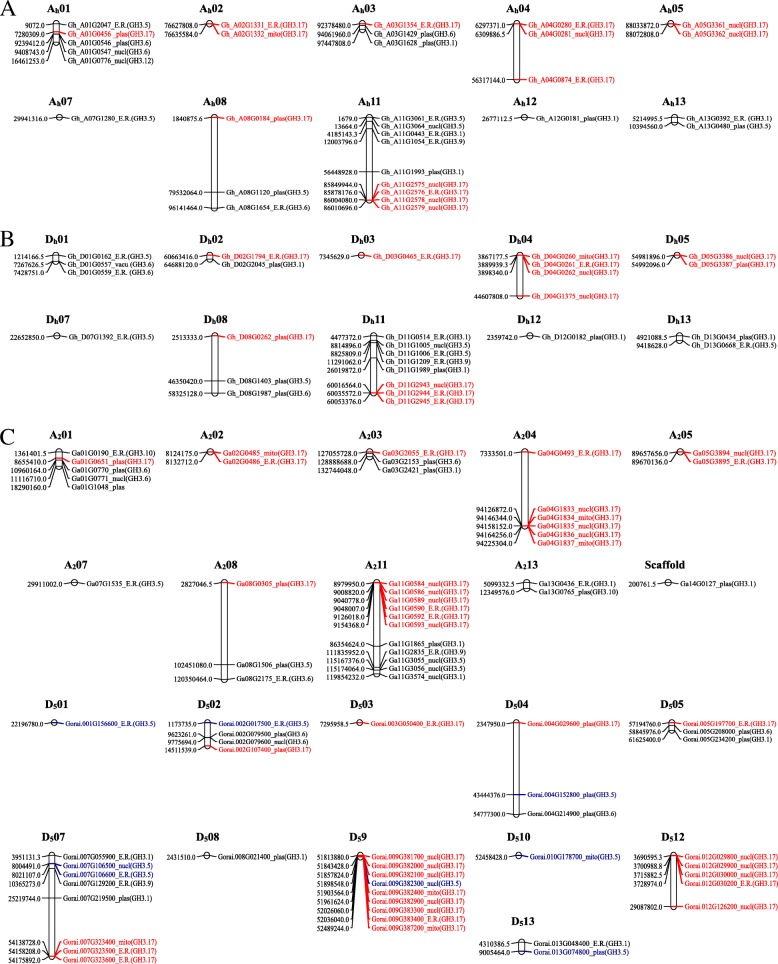
Chromosome mapping of the cotton *GH3* genes. **a**: chromosome mapping for the tetraploid cotton, *G. hirsutum*, **b**: chromosome mapping for diploid cotton, *G. raimondii* of the D genome; **c**: chromosome mapping for *G. arboreum* a diploid cotton of the A genome. Red colour shows the dominant members of the GH3 protein in AD genome, and the Blue colours illustrate the distribution of GH3.5 in A and D genomes

The *GH3* genes were not distributed in all the chromosomes; this was evident in all the three cotton species of AD, D, and A genomes. In the AD genome, the GH3 proteins were detected on 20 chromosomes out of the possible 26. Chromosome A_h_06, A_h_09 Ah10, and their homologs in the Dt-sub genomes were found to harbor no GH3 proteins. However, the highest gene loci were detected in chromosome A_h_01, A_h_03, A_h_04, A_h_08 and A_h_11 with 5, 3, 3, 3 and 9 genes, respectively. Moreover, higher proportions of *GH3* genes were also obtained for their homologous chromosomes in the Dt-sub genomes, with D_h_11 having (8 genes), D_h_01 (three genes), D_h_08 (three genes) and D_h_04 (four genes). The rest of the chromosomes of the AD genome harbored at least one (1) to a maximum of two (2) *GH3* genes. In *G. arboreum*, chromosome A_2_06 and A_2_10 harbored no genes, the highest gene loci were obtained in chromosome A_2_01, A_2_04, and A_2_11 with 5, 6 and 11 genes, respectively while the least gene loci were detected in chromosome A_2_07 with a single gene. Finally, gene mapping in *G. raimondii* of the D genome, no genes were found to be located in chromosome D_5_06 and D_5_11, but the highest gene loci were observed in chromosome D_5_07, D_5_09, and D_5_05, with 8, 9 and 5genes, respectively (Fig. 1). Moreover, we predicted the subcellular localization of the proteins encoded by the *GH3* genes among the proteins obtained from the tetraploid cotton. The majority of the proteins encoded by the *GH3* genes were found to be embedded with the endoplasmic reticulum (E.R) with 24 proteins accounting for 41.4%, followed by plasma membrane with 17, the nucleus with 14, mitochondrion with two and the vacuole with a single protein. In the two diploid cotton species, the highest proteins were found to be located within the nucleus with 12 and 11 proteins in *G. raimondii* and *G. arboreum*, respectively. The two cotton species harbored an equal number of GH3 proteins with 12 and 10 proteins predicted to be localized within the nucleus and the plasma membrane respectively. Moreover, variation was detected in the mitochondrion, with three (3) and four (4) GH3 proteins in *G. arboreum* and *G. raimondii*, respectively. The endoplasmic reticulum (E.R) plays an important role in enhancing stress tolerance through the presence of a mechanism that results from the accumulation of unfolded or misfolded proteins [45].

### Phylogenetic tree and gene structure analysis of the proteins encoded by the cotton *GH3* genes

**Fig. 2:**
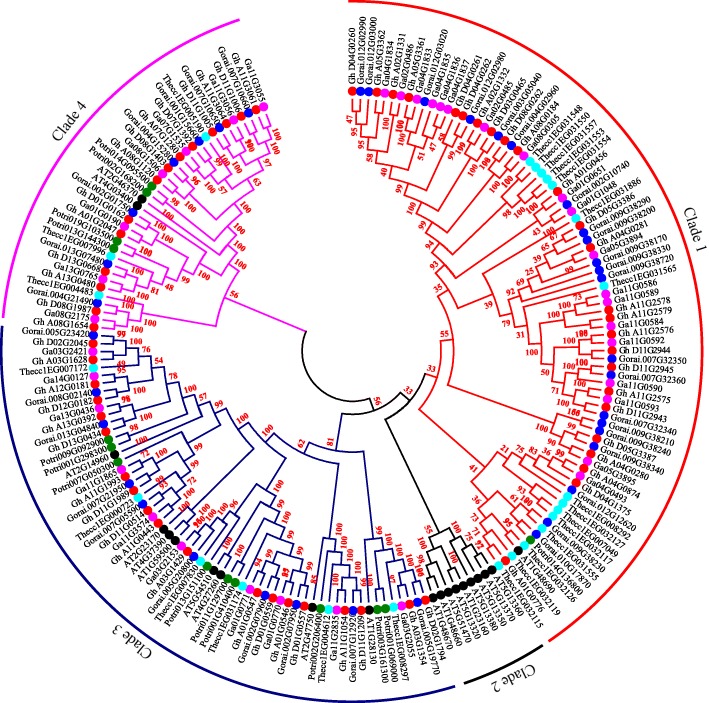
Phylogenetic tree of cotton GH3 proteins together with other plants

The GH3 proteins were phylogenetically classified into four main clades among all the three cotton species. Clade 1, clade 3 and clade 4 mainly contained the GH3 proteins from the three cotton species, except clade 2, which had mainly the GH3 proteins of the model plant, *Arabidopsis thaliana* (Fig. 2). In the clades, 3 and 4 in which the GH3 proteins from the three cotton species were located, no orthologous gene pairs existed except in clade 1, where orthologous genes were found between *Gorai.009G38230* and *Thecc1EG031555*; *Gorai.010G17860* and *Thecc1E032126*; and lastly *Gh_A01G0776* and *Thecc1EG032115*. The rest of the orthologous gene pairs were formed among the members of the cotton *GH3* genes. *Theobroma cacao* and *Gossypium* genus share a common ancestral background [46] and thus the detection of the orthologous gene pairs between the two species is appointer than the GH3 proteins are highly conserved. The entire cotton *GH3* genes in *G. hirsutum* (AD) and *G. arboreum* (A) genome, their gene structure was disrupted by introns, ranging from a minimum of 2 to a maximum of 8, for instance, in *G. hirsutum*, *Gh_D04G1375* and *Gh_D11G2945* contained the least number of introns (2), while *Gh_D08G0262* had the highest of eight (8) introns. Similarly, in *G. arboreum* of the A genome, nine genes harbored the least number of introns with three introns in each while two genes, *Ga01G1048* and *Ga08G0305* contained the highest number of introns with seven (7). Finally, in *G. raimondii* of the D genome, three genes were found to be intronless, *Gorai.009G382100*, *Gorai.009G382300* and *Gorai.010G178700*, while the rest harbored introns in the range of one (*Gorai.009G387200*) to a maximum of seven (*Gorai.004G029600*).

### Cis-regulatory element and GO analysis of the GH3 proteins of, *G. hirsutum*

**Fig. 3:**
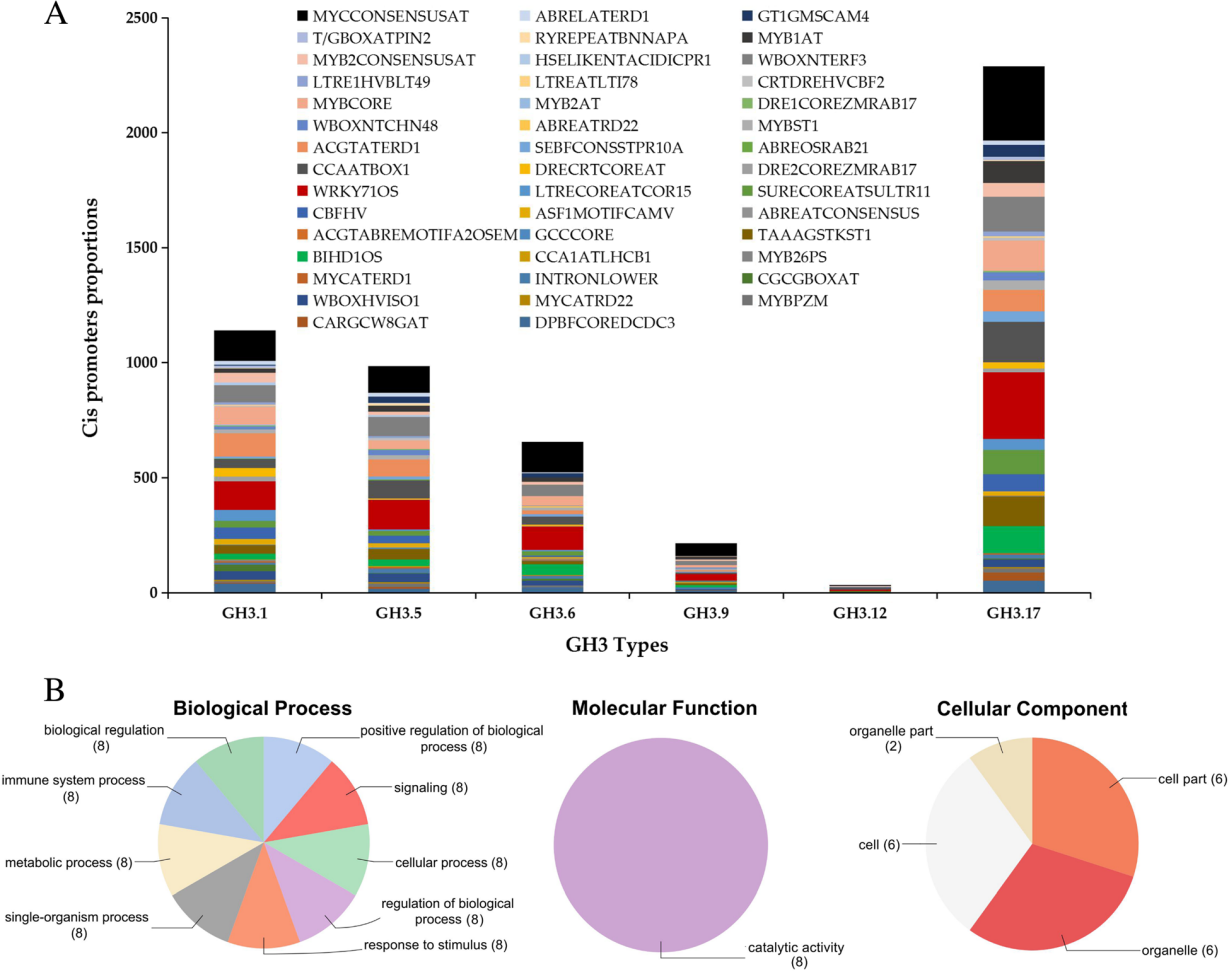
Cis-regulatory element analysis and GO functional annotation for the Upland cotton, *G. hirsutum GH3* genes. (**a**). Cis-regulatory element obtained in the analysis of the *GH3* genes in upland cotton; (**b**). Gene ontology functions detected for the various upland cotton *GH3* genes. The ordinate colours represent the proportions of various cis-regulatory elements determined for each of the six major groups of the cotton GH3 protein subfamilies

The various *GH3* genes in *G. hirsutum* were classified into six different types; *GH3.1*, *GH3.5*, *GH3.6*, *GH3.9*, *GH3.12* and *GH3.17*, in each gene type, the proportional number of the various cis-regulatory elements were varied, but averagely, the *GH3.17* harbored the highest number of the cis-regulatory elements while *GH3.12* had the least (Fig. 3a). The various cis-regulatory elements detected were; MYB2CONSENSUSAT (YAACKG) which functions in the MYB recognition site for the promoters of the dehydration-responsive gene; WBOXNTERF3 (TGACY) which could be involved in activation of *ERF3* gene by wounding; MYCCONSENSUSAT (CANNTG) involved in abiotic stress signaling in plant; ABREATRD22 (RYACGTGGYR) inducted by dehydration which is mediated by abscisic acid among others. ABA-responsive elements (ABRE) are among the top ranked cis-regulatory elements, which have been widely investigated in a number of plants; they are found to play a critical role in enhancing abiotic stress tolerance. In plants, the osmotic stress-responsive transcriptional regulation largely depends on two main types of the cis-regulatory elements, associated with the stress-responsive genes such as the ABREs and dehydration responsive elements (DREs). The DREs majorly are involved in the ABA independent pathway while ABRE is responsible for the detection of the ABA-mediated osmotic stress signals [47]. The proteins encoded by the *GH3* genes of the upland cotton were analyzed in order to determine if the genes could be associated with any of the gene ontological terms related to abiotic stress tolerance. GO delimits proteins encoded by the genes role were categorized into three; the cellular structural component (CC), molecular functions (MF) and biological processes (BP). Among the *GH3* genes obtained from the upland cotton, only 14 genes were found to have described GO terms. All the GO terms were detected, though the distributions were not symmetrical. Some of the genes harbored more of biological and molecular functions as opposed to cellular component roles; for instance, *Gh_D11G1005 (GH3.5)*, *Gh_D11G1006 (GH3.5)*, *Gh_A07G1280 (GH3.5)*, *Gh_A08G1120 (GH3.5)*, *Gh_D07G1392* (GH3.5), *Gh_D08G1403 (GH3.5)*, *Gh_A11G3064 (GH3.5)* and *Gh_A11G3061 (GH3.5)* were all found to have three biological processes and a single molecular function. These functions are jasmonic acid metabolic process (GO: 0009694), response to wounding (GO: 0009611), induced systemic resistance, jasmonic acid mediated signaling pathway (GO: 0009864) and jasmonate-amino synthetase activity (GO: 0080123). The rest of the genes had a cellular component function, chloroplast (GO: 0009507) among the rest except for *Gh_D11G1209 (GH3.9)* and *Gh_A11G1054 (GH3.9)* which were involved in chloroplast envelope (GO: 0009941) (Fig. 3b and Additional file 3: Table S3). The detection of jasmonic acid metabolic and signaling pathways is important since Jasmonic acid has been found to be an important plant hormone involved in the regulation of plant development and stress responses [48]. In the evaluation of the various cis-regulatory elements, several cis-regulatory elements were detected with a direct role in abiotic and biotic stress factors.

### RNA sequence analysis and relative transcriptome abundance in vegetative and reproductive tissues of *G. hirsutum* profiled under, drought and salt stress conditions

**Fig. 4:**
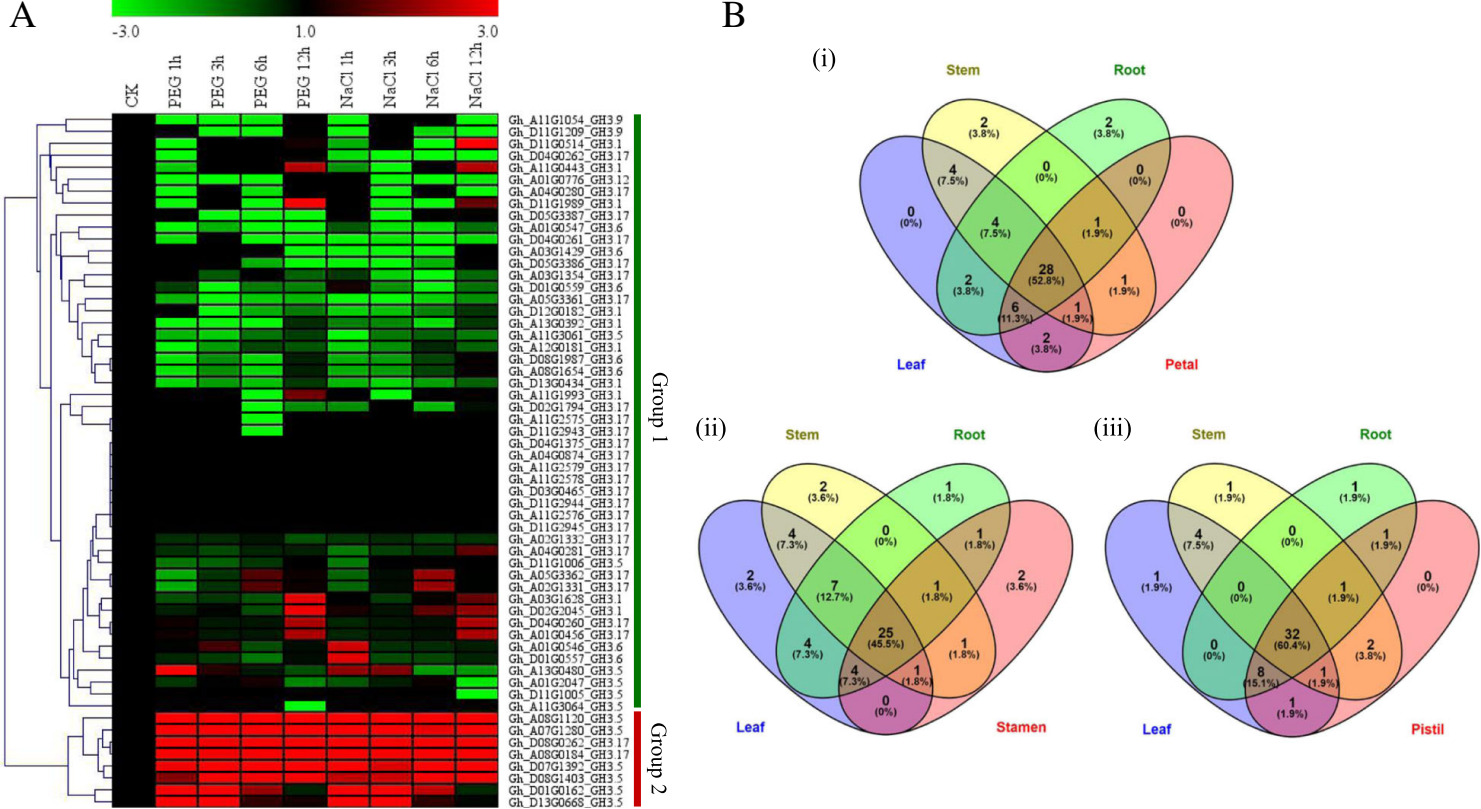
RNA sequence analysis and transcription abundance in vegetative and reproductive tissues of the upland cotton, *Gossypium hirsutum*. **a**: heat map; **b**: Venn diagram indicating tissues specific and common genes

In order to understand the possible role of the *GH3* genes in relation to abiotic stress factors. We obtained secondary RNA sequence data of the tetraploid cotton; *G. hirsutum*, *GH3* genes were profiled under salt and drought stress condition from the cotton functional genome database (https://cottonfgd.org/search/). The raw data were processed and their log 2 applied in the construction of the heatmap, in this analysis, only the leaf tissues were profiled at 1 h, 3 h, 6 h and 12 h of drought and salinity stress exposure. In the three stress levels, the expression pattern of the 58 *GH3* genes was subdivided into two levels, in level 1, a higher percentage of the genes were down-regulated or not expressed in various time points across the two stress factors, however, some of the genes exhibited up-regulation at specific time intervals. Intermittent type of gene induction was also observed under salt and drought stress conditions among the members of group1. Group 2 members were highly upregulated across the two stress levels, but of significance was *Gh_A08G1120 (GH3.5)* that was upregulated across the two stress factors, thus was considered to be of significance for further analysis (Fig. 4A). Its function and effects under drought and salt stress factors was further evaluated through RNAi by silencing the gene in *G. hirsutum*.

In the analysis of the abundance level of the transcription factors in the various tissues, leaf, stem, root and the three reproductive structures, petal, stamen (male part of the flower) and pistil (female part of the flower). Among the 58 *GH3* genes, various proportions were found to be present in the various tissues examined, for instance, only 47 *GH3* genes were found to be present in the leaf tissues, 41 in the stem, 43 in the root, 35 in the stamen, 39 in the petal and 46 were found to be inducted within the pistil. In evaluating the commonly inducted genes in various tissues, the vegetative tissues were compared to each of the three reproductive tissues. When the roots, stem, and leaves were compared to stamen, the tissues specific genes found were 2, 2, 1 and 2 genes in the leaf, stem, root and stamen tissues, respectively (Fig. 4B i). In relation to petal and pistil, no tissue specific genes were identified for the leaf and petal, when the three vegetative tissues were compared to the petal (Fig. 4B ii), indicating that all the genes induced at the petiolar region are similar to those inducted at the three vegetative tissues. Similar observations were noted among the genes inducted at the pistil regions, and were common to those inducted in the three vegetative tissues. However, in each of the vegetative tissues, there was a tissue specific gene, in each of the vegetative tissues, which was not among the genes induced at the pistil region (Fig. 4B iii). The common genes among the various vegetative tissues and the individual reproductive tissues were relatively higher, with proportions of 52.8, 45.5 and 60.4% common genes between the vegetative tissues and the reproductive tissues, petal, stamen and pistils respectively.

### Real time quantitative polymerase chain reaction (RT-qPCR) validation of the selected *GH3* genes

**Fig. 5:**
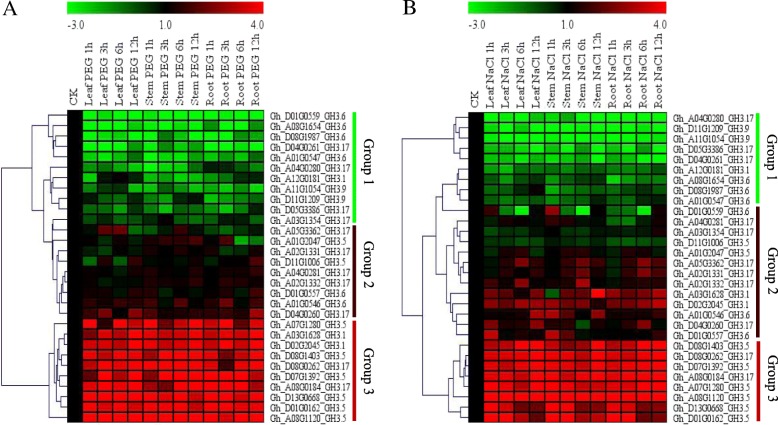
RT-qPCR analysis of the selected *GH3* genes under salt, drought and - stress conditions.  The heat map was visualized using the MeV_4_9_0 program. Red and green indicate high and low levels of expression, respectively. (**a**) Heat map showing 30 *GH3* genes profiled under drought stress, imposed by 17% of PEG-6000. (**b**) Heat map for the 30 *GH3* genes profiled under salt stress conditions, imposed by irrigating the upland cotton seedlings with 250 mM of NaCl solution

Due to the large numbers of *GH3* genes obtained from *G. hirsutum*, it was not possible to perform RT-qPCR for all the genes, a few numbers of them were selected for RT-qPCR validation. Since the RNA sequence revealed two groups based on their expression levels, only 30 genes were selected; top 10 significantly up-regulated genes, 10 differentially expressed and 10 down-regulated genes, the 30 genes selected were the representative of the entire *GH3* genes of *G. hirsutum*. Moreover, the thirty genes were common genes as per the Venn diagram analysis. The expression patterns of the genes were common across the two stress factors, drought, and salt stress. The drought stress was imposed by transferring the plants into Hoagland nutrient solutions, supplemented with 17% PEG-6000 in a hydroponic setup while salt stress was imposed by supplementing the Hoagland solution with 250 mM of sodium chloride solution. In all, the two stress factors, the 30 selected *GH3* gene expression patterns were, classified into three groups. The members of group 1, group 2 and group 3, exhibited a similar expression pattern all members of group 1 were down-regulated, group two showed differential expressions while group three members were significantly upregulated (Fig. 5a). A few numbers of genes were observed under salt stress conditions, out of 10 up-regulated genes, only eight genes showed significant up-regulation, the other two genes, *GhA03G1628 (GH3.1)* and *Gh_D02G2045 (GH3.1)* exhibited differential expression (Fig. 5b).

### Virus induced gene silencing (VIGS) confirmation by the expression analysis of *Gh_A08G1120 (GH3.5)* gene on tetraploid upland cotton

**Fig. 6:**
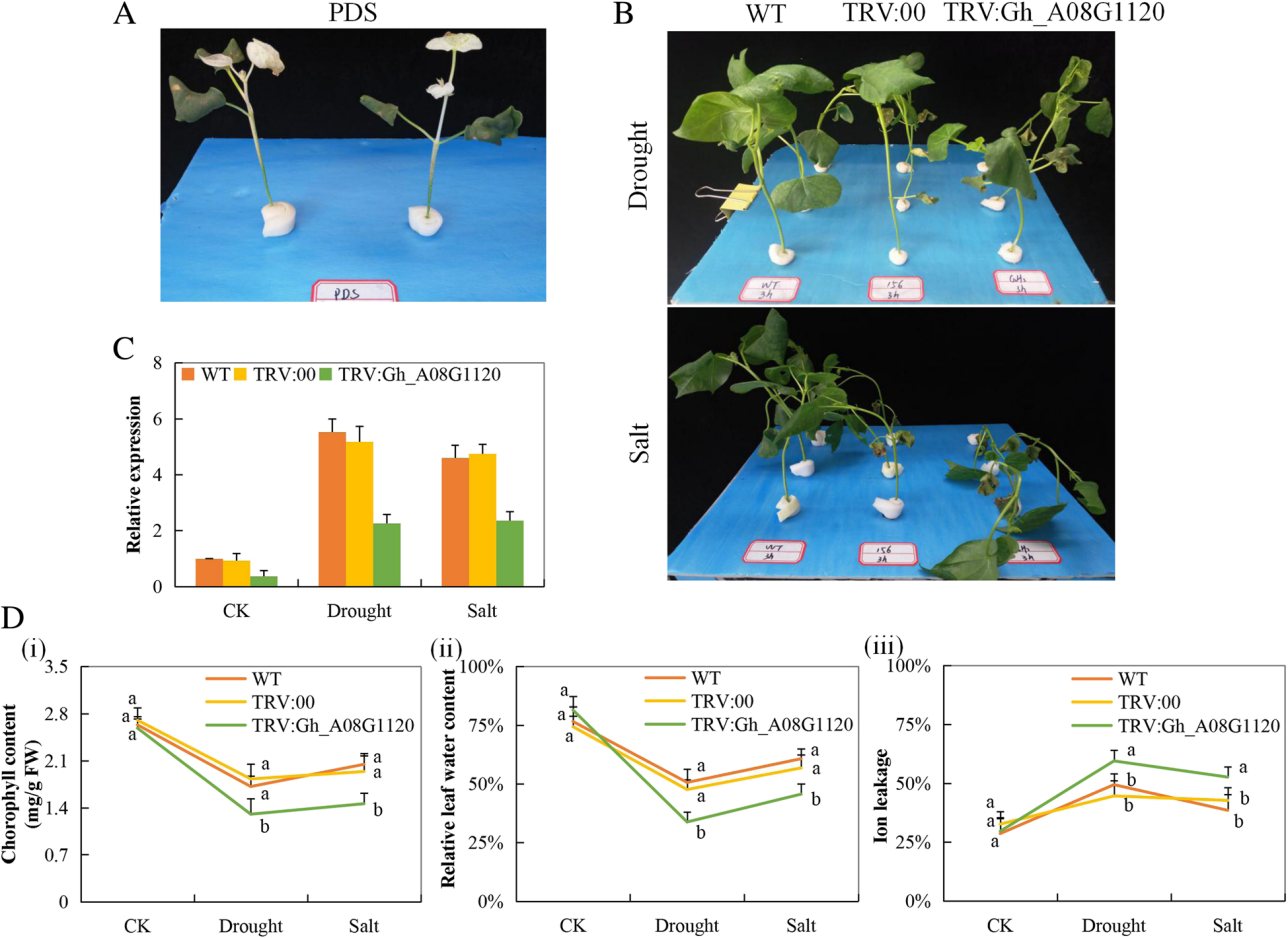
Phenotype trait evolution in the silenced plants with the TRV: 00 empty vector, wild type plants and *Gh_A08G1120 (GH3.5)* -silenced plants at 12 days post inoculation: (**A**). PDS infused plants (**B**). Drought and salt stress treatment. (**C**). RT-qPCR analysis of the change in the expression level of the *Gh_A08G1120 (GH3.5)* gene in cotton plants treated with VIGS. “TRV: 00” represents the plants carrying control the TRV2 empty vector; “TRV: *Gh_A08G1120 (GH3.5)”* represents the *Gh_A08G1120 (GH3.5)-*silenced plants. (**A**(**i**) Quantitative determination of chlorophyll content (**D**(**ii**)) Quantitative determination of relative leaf water content (RLWC) (**iv**) Quantitative determination of cell membrane stability (CMS) as ion leakage concentration in leaves of wild-type and *Gh_A08G1120 (GH3.5)* -silenced plants after 8-day post stress exposure Letters a/b indicate statistically significant differences (two-tailed, p < 0.01). In (**C** and **D**), each experiment was replicated three times. Bar indicates standard error (SE). Different letters indicate significant differences between wild type and OE lines (ANOVA; *p* < 0.05). CK: normal conditions

After seven days of post infiltration, the seedlings infiltrated with the vector containing the TRV: PDS showed albino like traits, white patches appeared on the first and subsequent leaves, after 20 to 25 days, the entire leaf surface was 100% bleached, and appeared white. The albino trait proved that the vector applied in silencing the novel gene was effective (Fig. 6A). The expression level of the novel gene in the various tissues of the VIGS and non-VIGS showed a significant difference, the expression level in the VIGS cotton tissues showed significant down-regulation of threefold compared to its expression in the non-silenced cotton seedlings (Fig. 6B-C). The significant differences in the expression pattern of the novel gene showed that its role had been highly reduced. The symptoms exhibited by the plants under various abiotic stress conditions, vividly indicated the inability of the plant to induct the silenced gene, thus the deleterious effects shown when exposed to abiotic stress conditions. Moreover, we evaluated the physiological traits of the VIGS and non-VIGS under abiotic stress conditions, the chlorophyll content, relative leaf water content (RLWC) and cell membrane stability (CMS) evaluated through ion leakage. The VIGS cotton seedlings exhibited significantly lower values of all the parameters measured compared to the wild plants under similar conditions (Fig. 6D i-iii). The reduction in all the traits measured showed that the silencing of the novel gene significantly reduced the ability of the plants to tolerate the effects caused by the various abiotic stress factors.

### Stress responsive transcript profiling and analysis of the oxidant-antioxidant enzymes on the tissues of VIGSVIGS and non-VIGS-cotton seedling exposed to drought and salt stress conditions

**Fig. 7:**
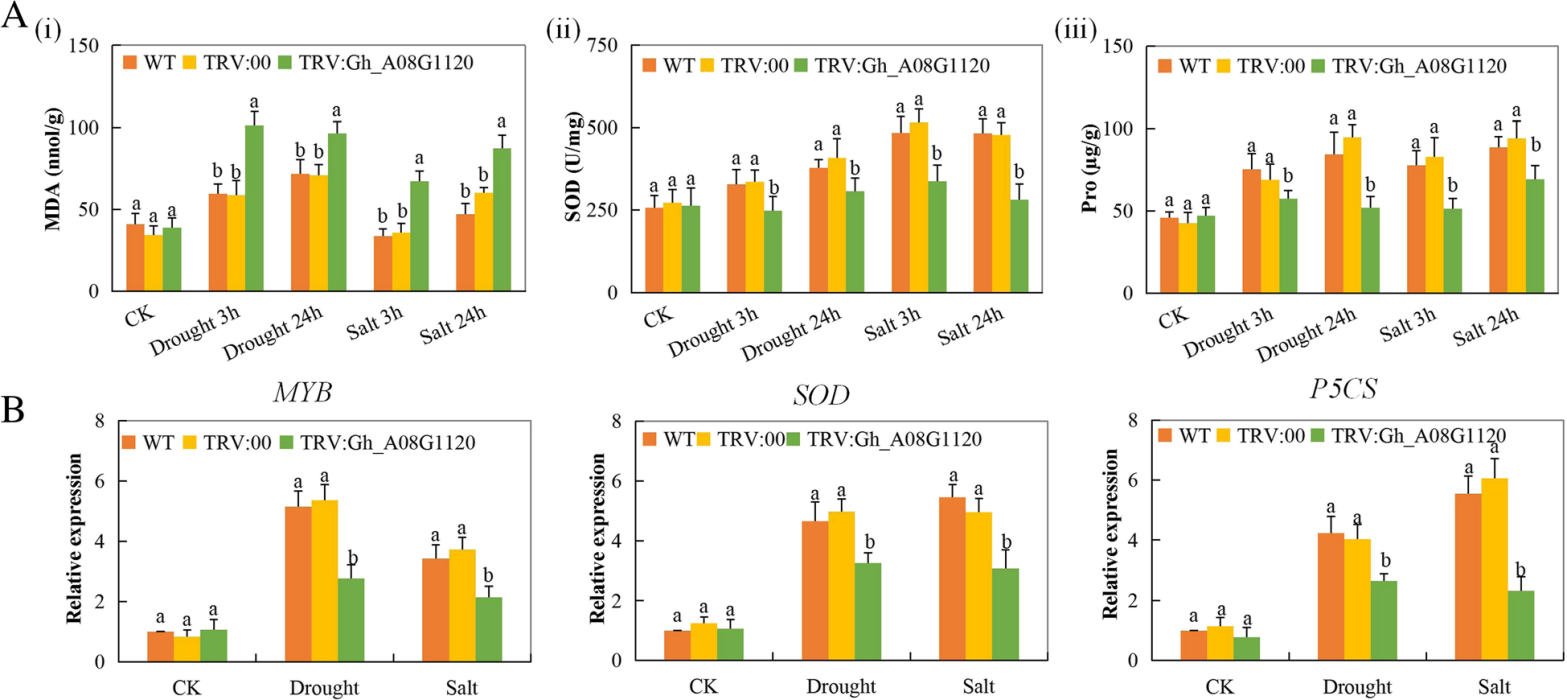
Determination of oxidants and antioxidants concentration levels in *Gh_A08G1120 (GH3.5)*-silenced plants and their wild types under drought and salt stress conditions: (**A** (**i**) Quantitative determination of malondialdehyde (MDA) (**A** (**ii**) Quantitative determination of SOD concentration. (**A** (**iii**) Quantitative determination of proline content in leaves of wild-type and VIGs plants after 8-day post stress exposure (**B**). RT-qPCR analysis of the change in the expression level of the *GhMYB, GhSOD* and *GhP5CS* stress resistant genes in cotton plants treated with VIGS. “TRV: 00” represents the plants carrying control the TRV2 empty vector, “TRV: *Gh_A08G1120 (GH3.5)*” represents the *Gh_A08G1120 (GH3.5) -*silenced plants. In each experiment was repeated three times. Bar indicates standard error (SE). Different letters indicate significant differences between wild type and OE lines (ANOVA; *p* < 0.05). CK: indicate normal conditions

In this work, proline levels, Superoxide dismutase (SOD) and malondialdehyde (MDA) were evaluated. MDA concentration levels were higher in the leaf tissues of the VIGS cotton seedlings whereas the SOD and proline concentration levels were significantly reduced. Moreover, the SOD and proline concentration levels were significantly higher in the leaves of the wild type compared to the VIGS cotton exposed to drought and salt stress conditions (Fig. 7A i-iii). The SOD concentration level was significantly lower, an indication that the VIGS plants suffered extensive oxidative damage, which was further evidenced by the high concentration levels of MDA. When plants are subjected to dehydration stress and change in osmotic pressure they accumulate solutes such as amino- acids, and sugars [49]. In our study, there was an increase in proline concentration. Proline contributes to osmotic adjustment, detoxification of ROS, and protection of membrane integrity hence, tolerance to drought and salt stresses. From previous research studies, proline has been observed to act as an osmolyte, a signaling molecule and an antioxidative defense molecule [50]. Three stress responsive genes, *GhMYB, GhSOD*, and *GhP5CS* were used for evaluating the effect of suppression of the novel gene in cotton under drought and salt stress conditions. In all the three stress responsive genes, their expression levels were found to be significantly reduced in the VIGS plants compared to wild types (Fig. 7B). The down-regulation of these genes showed the plants were highly susceptible to drought and salt stress effects and their ability to tolerate the various abiotic stress factors were significantly reduced, thus causing a higher oxidative injury.

## Discussion

The *GH3* genes play an integral hormonal role in regulating growth and development; it functions in homeostasis by conjugation of amino acids of plant growth regulators such as jasmonic acids. These genes are stimulated rapidly in the primary response of plants to auxins. The *GH3* family genes, have been identified and functionally studied in several plant species, which includes; tomatoes [51], legumes [52], *Medicago truncatula* [24], apple [19], Arabidopsis [53], rice [26, 54] and even in soybean [55] among others. In the study of the *GH3* family in the three cotton species, *G. hirsutum, G. raimondii* and *G. arboreum*, the protein PFAM domain, PF03321, was used, in which 58, 38 and 36 proteins encoded by the *GH3* genes were obtained from *G. hirsutum, G. arboreum* and *G. raimondii,* respectively. We observed that there was an element of gene loss evident in the less number of genes in *G. hirsutum*, which is an upland cotton with 58 genes as compared to *G. raimondii* and *G. arboreum* having 38 and 36 genes, respectively. Gene loss occurs when there is chromosome doubling during polyploidization and rearrangement of genomic sequences after hybridization [56]. The results obtained showed that the proteins encoded by the *GH3* genes in cotton were higher compared to other plants such as chickpea with 11, soybean with 28, Lotus with 18, Medicago with 10 and apple with *GH3* genes.

In the tetraploid cotton, *G. hirsutum* the genes were distributed in only 20 out of the 26 chromosomes.Ah06, Ah09 and Ah10, and their homologs in the Dt-sub genomes were found to harbor no GH3 proteins. The GH3 proteins existed in five group members, namely GH3.1, GH3.4, GH3.6, GH3.9 and GH3.17 that were common among the three cotton species; however, in *G. hirsutum* there was GH3.12 while *G. arboreum* had an extra group member GH3.10. The highest number of members of the *GH3* gene family was the GH3.17 with 26, 20, and 20 genes in *G. hirsutum, G. arboreum* and *G. raimondii* respectively, from previous studies *AtGH3.17* was observed to display enzymatic activity with IAA in Arabidopsis [57]. The majority of the GH3 proteins were embedded in the endoplasmic reticulum, followed by the nucleus and plasma membrane, the accumulation of unfolded/misfolded proteins in the ER activates the unfolded protein response (UPR), the UPR is closely related to heat stress, drought stress and salt stress in plants [58]. The nucleus coordinated functions, and interactions are important in the stress response, their functions towards abiotic stress are based on transcriptional regulation, signaling, and gene regulation [59]. The plasma membrane, on the other hand, play an important role in the exchange of compounds such as metal ions, metabolites, and nutrients, hence it controls the influx of ions in the cell when the plant faces abiotic stresses such as drought [60]. From the phylogenetic analysis, there was a close relationship between *Theobroma cacao* and *Gossypium* genus that existed between clades 3 and 4, which suggested that the functions of the *GH3* genes in cotton were similar to the *GH3* genes in cacao plant species, hence they share a common ancestry [61]. Though the orthologous pair existed between different species of cotton, it was observed that the pair were members of the same gene family, for example *Gh_A08G1120* was orthologous to *Ga08G1560* and both were members of the GH3.5 proteins, *Gh_A03G1628* was orthologous to *Ga03G2421* both the members of the GH3.1 proteins. Studying the homologous genes is important, because the same genes in two different species (orthologs) are more likely to have the same cellular function than two duplicated genes (paralogs) [62]. All the genes were disrupted by introns with *Gh_D08G0262* having the highest eight (8) introns. Previous studies revealed that introns lacked function [63], hence their existence on transcribed gene parts that are free from selective constraints, triggered an increase in genetic diversity that eventually led to the gain of many introns-related functions, the presence of introns is believed to be so efficient in boosting expression levels [64]. In *G. raimondii* three genes were found to be intronless, some studies show that introns must have existed in prokaryotes, only to be later eliminated completely from their genomes due to the genome streamlining hence lack of introns in some of the present genes [65].

All the three GO term components were observed, however, not all the genes classified under this family had described GO functions. In upland cotton, only 14 genes accounting for 24.1% were found to have described GO terms, similar results were observed by [66] were 33.7% of GH3 rice proteins from TIGR showed no Gene Ontology (GO) assignation. Most of the genes were observed to be performing biological processes; biological processes are regulated by several means this includes the control of gene expression, protein modification or interaction with a protein or substrate molecule [67]. The detection of jasmonic acid pathways and signaling plays an important role in understanding the role in which this plant hormone could be playing in abiotic stress conditions, similar results were noted in [68] where 3 GH3 proteins had important roles in jasmonic acid adenylation. We performed cis-regulatory element analysis and identified cis-regulatory elements that performed the regulatory function towards abiotic stress conditions; we found out that 44 cis-regulatory elements had a regulatory function toward abiotic stress with the highest cis-regulatory element being MYCCONSENSUSAT, which performs the function of specificity in abiotic stress signalling in plants. Most of the cis-regulatory elements were harbored in *GH3.17* gene types with a descriptive role as an Indole-3-acetic acid-amido synthetase, the *GH3.17* gene has been found to display enzymatic activity in IAA in Arabidopsis [24]. Indole-3-acetic acid-amido synthetase was found to be suppressed in above ground tissues in rice, but it’s dramatically expressed when the plant is exposed to drought stress conditions [69]. By the use of the RNA, sequence data of *G. hirsutum* it was observed that some genes exhibited differential expression while others were upregulated at specific time intervals, this trend was also observed under different stress treatment. The *Gh_A08G1120 (GH3.5)* was of significance, since it was upregulated in all the stress conditions, hence the choice of this gene for further analysis. We conducted RT-qPCR analysis to validate the effects drought and salt stress conditions on the *GH3* genes, it was noted that among the stress condition, more *GH3* genes were highly upregulated under drought conditions as compared to the salt condition. It was observed that all the genes that showed higher expression under salinity stress also showed similar expression patterns under drought conditions. Interestingly the gene *Gh_A08G1120 (GH3.5)* remained highly expressed under drought and salt conditions; this could possibly show significant biological roles this gene could be playing in enhancing tolerance towards salt and drought stresses in the cotton plant. The GH3.5 has been associated with plant hormone signal transduction function, Zhang et al. [70] on the experiment to determine the role it performed in Arabidopsis it was noted that it acted as bi-functional modulator in both SA and auxin signaling during pathogen infection. Moreover, a homeolog form of *GH3.5* gene, *WES1*, has been to be strongly induced by Salicylic acid (SA) and pathogen infections. Furthermore, the mutant form *wes1-D* showed reduced growth and highly susceptible to pathogen infections. But more importantly, the *WES1*-overexpressed plants registered higher concentration levels of ABA, an indication that *WES1* would also be involved in ABA-regulated abiotic stress responses [53]. We then silenced the *Gh_A08G1120 (GH3.5)* gene in upland cotton to determine the effects on the downregulation of the gene of the VIGS-plants compared to their wild types. The TRV: PDS infused plants showed an albino like traits on the first foliage leaves after seven days of post-inoculation. The RT-qPCR analysis was then performed to evaluate the effect of gene silencing; the expression level of the *Gh_A08G1120 (GH3.5)* gene was significantly reduced in the *Gh_A08G1120 (GH3.5.)*-silenced plants than in the TRV: 00 and or the wild types plants, under drought and salt stress conditions. The knockdown of the *Gh_A08G1120 (GH3.5)* gene in cotton compromised the plant′s ability to tolerate drought and salt stresses; this confirmed the significance of this gene in enhancing drought and salt stress tolerance in cotton. We then explored the mechanism of this gene towards stress by analyzing physiological and biochemical parameters, chlorophyll content, the presence or absence of oxidant and antioxidant enzymes, such as proline, superoxide dismutase (SOD) and malondialdehyde (MDA).

The chlorophyll content and activities of SOD were significantly reduced in the *Gh_A08G1120 (GH3.5)* gene silenced plants while proline and MDA were found to have higher levels. The induction of salt and drought stress to the plants resulted in the plant overproduction of reactive oxygen species (ROS) this lead to oxidative destruction to plant cell structures and its components, and finally death of the plant. The higher levels of proline and MDA suggest that the silenced plants had a reduced ability to properly to scavenge on the ROS leading to the destruction of the cell membrane and reduction of chlorophyll content. Moreover, all the stress responsive genes, *GhMYB, GhSOD* and *GhP5CS*, were all downregulated in the leaf tissue of the *GH3*-silenced plants, but were upregulated in the wild plants under drought and salt stress conditions. The downregulation of the known stress responsive genes in the *Gh_A08G1120 (GH3.5)* Knocked plants showed that the proteins encoded by the gene had a significant role in enhancing plants response to either drought and or salt stress. Moreover, analysis of *AtGH3.5* indicated, that this protein can conjugate with SA but is more efficient using benzoic acid (BA) as a substrate [71]. In plants, SA is a major phytohormone that mediates plant disease resistance and abiotic stress responses, and overexpression of *AtGH3.5* in Arabidopsis led to changes in SA levels in some overexpressing transgenic plants [72]. All plants have different ways of oxidizing the reactive oxygen species (ROS), including antioxidant enzymes, such as superoxide dismutase (SOD), catalase (CAT), peroxidase (POD), ascorbate peroxidase (APX), among other [73]. SOD enzyme is one of the most important antioxidants which oxidizes the reactive oxygen species leading to the formation of hydrogen peroxide and oxygen, which are less toxic to the plant cells [74]. The downregulation of the *SOD* gene in the tissues of the VIGS cotton showed that the plants had lost the ability to scavenge on the ROS, an indication that the cotton *GH3* gene has a vital role in plants under abiotic stress condition.

## Conclusions

In this research work, we carried out genome-wide identification and functional characterization of the *GH3* genes in cotton, and identified a total of 132 proteins encoded by the *GH3* genes, with 58, 38 and 36 GH3 proteins in *G. hirsutum, G. raimondii* and *G. arboreum*. The genes were found to be distributed across the various cotton chromosomes, though with asymmetrical distribution pattern. In the determination of subcellular localization of the proteins encoded by the *GH3* genes, endoplasmic reticulum, nucleus and plasma membrane were the major cellular structures with the highest proportions of the GH3 proteins across the three cotton species. The endoplasmic reticulum is an important plant organelle, and it has been found that regulated intramembranous proteolysis triggered by E.R stress mediates some forms of stress signaling and in turn enhances stress acclimation in Arabidopsis [75]. Therefore, the higher proportions of the GH3 proteins in E.R could be integral for the detection of stress signals in cotton. Moreover, in the functional characterization of the *GH3* genes in cotton, showed that knockdown of *Gh_A08G1120 (GH3.5)* highly compromised the ability of cotton plants to tolerate drought and salt stress as evident by a high level of oxidant enzyme, MDA and significant reduction in the levels of antioxidants, proline and SOD. In addition, the stress responsive genes were all down-regulated in the tissues of the *Gh_A08G1120 (GH3.5)-*silenced plants, but were highly up-regulated on the tissues of the wild plants under drought and salt stress conditions. Furthermore, plants need accurate control mechanism over growth regulators during growth and development, in addition to their responses to both biotic and abiotic stress factors. In order to ensure precise control and coordination, plants adopt the modulation of the active plant hormones through conjugation of the bioactive phytohormone molecules to amino acids through acyl acid amido synthetases of the GH3 protein family. Moreover, studies have shown that the GH3.5 protein of the model plant, Arabidopsis conjugates several molecules from anumber of phytohormone pathways thereby enhancing their response to abiotic and biotic stress factors. The results provide a solid foundation through which the molecular functions of the GH3 proteins in cotton can be further explored in order to know the exact role played by the proteins encoded by the *GH3* genes.

## Additional files

Additional file 1: **Table S1.** Primers details used for RT-qPCR analysis of the *Gossypium hirsutum GH3* genes under drought and salt stress conditions. (DOCX 18 kb)

Additional file 2: **Table S2.** physiochemical properties of the proteins encoded by the cotton *GH3* genes. (DOCX 78 kb)

Additional file 3: **Table S3.** Go analysis of the upland cotton, *G. hirsutum GH3* genes. (DOCX 21 kb)

## Data Availability

All the relevant data and Additional files are all availed including the sequences of the primers used in the *GH3* genes expression profiling.

## Abbreviations

GO: Gene ontology
MDA: Malondialdehyde
POD: Peroxidase
ROS: Reactive oxygen species
SOD: Superoxide dismutase
TRV: Tobacco rattle virus
