# Supplementary material for: Functional characterization of Gh_A08G1120 (GH3.5) gene reveal their significant role in enhancing drought and salt stress tolerance in cotton
Source: BMC Genet. 2019 Jul 23;20:62. doi: 10.1186/s12863-019-0756-6 (PMC6651995; doi:10.1186/s12863-019-0756-6)
Supplement: Supplementary file 1 — Table S1. Primers details used for RT-qPCR analysis of the Gossypium hirsutum GH3 genes under drought and salt stress conditions. (DOCX 18 kb) [file 12863_2019_756_MOESM1_ESM.docx]

Table S1: primer details

| Gene ID | Forward Sequence | Reverse sequence |
| --- | --- | --- |
| Gh_A01G0546 | AGTGAACCATTTGCTGCCCTTT | CCCAGTACAGCACATAGTGGC |
| Gh_A01G0547 | CACGCAATGCTCATGTTGAGT | GGCAATACGGTCAATATGGGG |
| Gh_A01G2047 | AGCTGAAGCTAGCAATGGCA | GGTGTCGAACCAGCGGATAA |
| Gh_A02G1331 | AGCTGAACCCATTACAGGTTCTAT | TGAAGTCCCAGAGCTGCATT |
| Gh_A02G1332 | AGCTGAACCCATTACAGGTTCTAT | AAGTCCCAGAGCTGCATTTGA |
| Gh_A03G1354 | TCGTGCATCGGAGGAATGTC | CCTTTGCCTTTGTCACTGCC |
| Gh_A03G1628 | CCAACGCATCGCTAATGGTG | GCGGCGATCTAGCTCTTCTT |
| Gh_A04G0280 | AAGCCCCACCTTCAAAACCA | CCTCGTGTCGTCGGATTAGG |
| Gh_A04G0281 | AGCTATGCGGAGACATCATCG | CTTGGCATCAAGTTTTGGGCA |
| Gh_A05G3362 | AATGCAGCTCTGGGACTTCA | TCAGCAGTGACAGGCATCAG |
| Gh_A07G1280 | GAAGCGCCGAGTATTTGCAG | GGGAAGAAGCACCATCGACA |
| Gh_A08G0184 | TGTTTGATTAAATGCAGCTCTGGG | ATGTCGCCGTTATGTGAGCA |
| Gh_A08G1120 | GGGACTACTCAAGGAAAGCCC | TCTCAATAGGAAATTCACACCTTGT |
| Gh_A08G1654 | CACCTCTTGTCCGTTGTGT | ATATGCGACGCCCCTTTTGA |
| Gh_A11G1054 | TAACGACGACAGCTGGTGAA | AGGTAATAACCGGGACACAACT |
| Gh_A12G0181 | TCCCTCGTGATTCACCTCCT | CGGTACCTGTACAACCCTGC |
| Gh_D01G0162 | TTCGGGTTCGGGTAGTGTTG | GACTTGGGAGAGTGGAACCG |
| Gh_D01G0557 | CTCATGTTGAGTACCTGCAAACAC | TGGCAATACGGTCAACATAAGG |
| Gh_D01G0559 | CCATTTGCTGCCCTTTGGTG | GTGGCCTGGAATTGTTGTGG |
| Gh_D02G2045 | AAATGACTCGGAACGCCGAC | CGATCGCTTGCCCCATTGAG |
| Gh_D04G0260 | TCCTCCATGGTCAAACCGAG | CTGAAGTCCCAGAGCCTGTA |
| Gh_D04G0261 | TCAAGACGAGGAATTGGGGG | TGACCATGGAGGAATCCCCT |
| Gh_D05G3386 | AGTGCGCCTAAAATCTGCAC | ACTTGGTGAAATTTGCTTGTGGT |
| Gh_D07G1392 | GAAGCGCCGAGTATTTGCAG | GAATCGGGGAAGAAGCACCA |
| Gh_D08G0262 | GAACCCCTCACTGGCTCTG | GTAGGTACCAGCTTCGGCAG |
| Gh_D08G1403 | TAGGGTTAGGAGCTGCCCTT | GTAGACCCTACACATCGCGG |
| Gh_D08G1987 | GTGTCACGACCGGGAGTATG | TAATGGCCACGCAACACTCT |
| Gh_D11G1006 | AAATCTGGGCCTCAATGGCA | CCGGTGAGAATCGGGGAAAA |
| Gh_D11G1209 | CAGCCAAGGGGGCTCAATTA | AGCCATTCAAGGCAACCCAT |
| Gh_D13G0668 | AGGAGAGGGTGCTAGGTGAG | CCTGGACACAACATAGCCGT |
