# Supplementary material for: Functional characterization of Gh_A08G1120 (GH3.5) gene reveal their significant role in enhancing drought and salt stress tolerance in cotton
Source: BMC Genet. 2019 Jul 23;20:62. doi: 10.1186/s12863-019-0756-6 (PMC6651995; doi:10.1186/s12863-019-0756-6)
Supplement: Supplementary file 2 — Table S2. physiochemical properties of the proteins encoded by the cotton GH3 genes. (DOCX 78 kb) [file 12863_2019_756_MOESM2_ESM.docx]

Table S2: physiochemical properties of the *GH3* genes in *Gossypium hirsutum*, *Gossypium raimondii* and *Gossypium arboreum*

| **Cotton Genome** | **Protein ID** | **Gene Name** | **Description** | **Chro.** | **Start** | **End** | **Strand** | **Length (bp)** | **Transcript Length (bp)** | **CDS Length (bp)** | **CDS GC Content (%)** | **Exon Number** | **Mean Exon Length (bp)** | **Mean Intron Length (bp)** | **Protein Length (aa)** | **Molecular Weight (kDa)** | **Charge** | **pI** | **GRAVY** |
| --- | --- | --- | --- | --- | --- | --- | --- | --- | --- | --- | --- | --- | --- | --- | --- | --- | --- | --- | --- |
| **AD** | Gh_A01G0456.1 | GH3.17 | Indole-3-acetic acid-amido synthetase GH3.17 | A01 | 7,278,255 | 7,282,362 | + | 4,108 | 1,566 | 1,566 | 42.1 | 5 | 313.2 | 635.5 | 521 | 58.269 | -0.5 | 6.408 | -0.051 |
|  | Gh_A01G0546.1 | GH3.6 | Indole-3-acetic acid-amido synthetase GH3.6 | A01 | 9,238,374 | 9,240,450 | + | 2,077 | 1,842 | 1,842 | 43.8 | 3 | 614 | 117.5 | 613 | 69.496 | -2 | 6.207 | -0.28 |
|  | Gh_A01G0547.1 | GH3.6 | Indole-3-acetic acid-amido synthetase GH3.6 | A01 | 9,407,662 | 9,409,824 | + | 2,163 | 1,842 | 1,842 | 43.5 | 3 | 614 | 160.5 | 613 | 69.261 | -0.5 | 6.43 | -0.284 |
|  | Gh_A01G0776.1 | GH3.12 | 4-substituted benzoates-glutamate ligase GH3.12 | A01 | 16,460,997 | 16,461,509 | - | 513 | 345 | 345 | 42.6 | 3 | 115 | 84 | 114 | 13.01 | 7.5 | 10.16 | -0.432 |
|  | Gh_A01G2047.1 | GH3.5 | Probable indole-3-acetic acid-amido synthetase GH3.5 | A01 | 7,113 | 11,031 | + | 3,919 | 1,809 | 1,809 | 44.6 | 4 | 452.3 | 703.3 | 602 | 68.687 | 6.5 | 7.326 | -0.298 |
|  | Gh_A02G1331.1 | GH3.17 | Indole-3-acetic acid-amido synthetase GH3.17 | A02 | 76,626,585 | 76,629,036 | - | 2,452 | 1,755 | 1,755 | 39.9 | 4 | 438.8 | 232.3 | 584 | 66.037 | 2.5 | 7.001 | -0.179 |
|  | Gh_A02G1332.1 | GH3.17 | Indole-3-acetic acid-amido synthetase GH3.17 | A02 | 76,634,021 | 76,637,141 | - | 3,121 | 1,758 | 1,758 | 40.3 | 4 | 439.5 | 454.3 | 585 | 66.14 | 1 | 6.693 | -0.175 |
|  | Gh_A03G1354.1 | GH3.17 | Indole-3-acetic acid-amido synthetase GH3.17 | A03 | 92,377,252 | 92,379,703 | - | 2,452 | 1,935 | 1,935 | 42.4 | 5 | 387 | 129.3 | 644 | 73.211 | 0 | 6.511 | -0.284 |
|  | Gh_A03G1429.1 | GH3.6 | Indole-3-acetic acid-amido synthetase GH3.6 | A03 | 94,060,935 | 94,062,983 | + | 2,049 | 1,848 | 1,848 | 43.2 | 3 | 616 | 100.5 | 615 | 69.133 | -5.5 | 5.339 | -0.174 |
|  | Gh_A03G1628.1 | GH3.1 | Probable indole-3-acetic acid-amido synthetase GH3.1 | A03 | 97,446,817 | 97,448,799 | - | 1,983 | 1,797 | 1,797 | 49 | 3 | 599 | 93 | 598 | 67.903 | 3 | 7.011 | -0.321 |
|  | Gh_A04G0280.1 | GH3.17 | Indole-3-acetic acid-amido synthetase GH3.17 | A04 | 6,295,356 | 6,299,386 | + | 4,031 | 1,764 | 1,764 | 41.8 | 4 | 441 | 755.7 | 587 | 66.898 | -6 | 5.37 | -0.219 |
|  | Gh_A04G0281.1 | GH3.17 | Indole-3-acetic acid-amido synthetase GH3.17 | A04 | 6,308,669 | 6,311,104 | + | 2,436 | 1,695 | 1,695 | 41.1 | 4 | 423.8 | 247 | 564 | 63.231 | -2 | 6.141 | -0.168 |
|  | Gh_A04G0874.1 | GH3.17 | Indole-3-acetic acid-amido synthetase GH3.17 | A04 | 56,316,128 | 56,318,166 | - | 2,039 | 1,629 | 1,629 | 41.1 | 5 | 325.8 | 102.5 | 542 | 61.207 | -1.5 | 6.26 | -0.149 |
|  | Gh_A05G3361.1 | GH3.17 | Indole-3-acetic acid-amido synthetase GH3.17 | A05 | 88,032,832 | 88,034,906 | + | 2,075 | 1,773 | 1,773 | 41.1 | 4 | 443.3 | 100.7 | 590 | 66.558 | -4.5 | 5.633 | -0.169 |
|  | Gh_A05G3362.1 | GH3.17 | Indole-3-acetic acid-amido synthetase GH3.17 | A05 | 88,042,232 | 88,103,387 | + | 61,156 | 1,758 | 1,758 | 40.3 | 4 | 439.5 | 19,799.30 | 585 | 66.189 | -4 | 5.656 | -0.209 |
|  | Gh_A07G1280.1 | GH3.5 | Probable indole-3-acetic acid-amido synthetase GH3.5 | A07 | 29,939,596 | 29,943,031 | - | 3,436 | 1,758 | 1,758 | 44.1 | 4 | 439.5 | 559.3 | 585 | 65.974 | -3.5 | 6.083 | -0.178 |
|  | Gh_A08G0184.1 | GH3.17 | Indole-3-acetic acid-amido synthetase GH3.17 | A08 | 1,836,691 | 1,845,060 | - | 8,370 | 3,354 | 3,354 | 40.8 | 8 | 419.3 | 716.6 | 1,117 | 124.546 | -1 | 6.427 | -0.147 |
|  | Gh_A08G1120.1 | GH3.5 | Probable indole-3-acetic acid-amido synthetase GH3.5 | A08 | 79,530,978 | 79,533,142 | - | 2,165 | 1,719 | 1,719 | 43.2 | 4 | 429.8 | 148.7 | 572 | 64.383 | -3 | 6.049 | -0.145 |
|  | Gh_A08G1654.1 | GH3.6 | Probable indole-3-acetic acid-amido synthetase GH3.6 | A08 | 96,140,440 | 96,142,485 | - | 2,046 | 1,731 | 1,731 | 41.7 | 3 | 577 | 157.5 | 576 | 64.623 | 3.5 | 6.962 | -0.116 |
|  | Gh_A11G0443.1 | GH3.1 | Probable indole-3-acetic acid-amido synthetase GH3.1 | A11 | 4,184,163 | 4,186,123 | + | 1,961 | 1,779 | 1,779 | 45.6 | 3 | 593 | 91 | 592 | 67.383 | -6 | 5.569 | -0.208 |
|  | Gh_A11G1054.1 | GH3.9 | Putative indole-3-acetic acid-amido synthetase GH3.9 | A11 | 12,002,212 | 12,005,380 | + | 3,169 | 1,788 | 1,788 | 44 | 4 | 447 | 460.3 | 595 | 67.035 | -5.5 | 5.565 | -0.131 |
|  | Gh_A11G1993.1 | GH3.1 | Probable indole-3-acetic acid-amido synthetase GH3.1 | A11 | 56,447,917 | 56,449,936 | - | 2,020 | 1,821 | 1,821 | 46.9 | 3 | 607 | 99.5 | 606 | 68.574 | -5.5 | 5.589 | -0.215 |
|  | Gh_A11G2575.1 | GH3.17 | Indole-3-acetic acid-amido synthetase GH3.17 | A11 | 85,848,892 | 85,850,989 | + | 2,098 | 1,755 | 1,755 | 40.3 | 4 | 438.8 | 114.3 | 584 | 66.123 | -6.5 | 5.158 | -0.143 |
|  | Gh_A11G2576.1 | GH3.17 | Indole-3-acetic acid-amido synthetase GH3.17 | A11 | 85,877,033 | 85,879,316 | + | 2,284 | 1,677 | 1,677 | 40.5 | 4 | 419.3 | 202.3 | 558 | 62.6 | -2 | 5.713 | -0.066 |
|  | Gh_A11G2578.1 | GH3.17 | Indole-3-acetic acid-amido synthetase GH3.17 | A11 | 86,002,957 | 86,005,198 | - | 2,242 | 1,743 | 1,743 | 40.1 | 4 | 435.8 | 166.3 | 580 | 65.525 | -5.5 | 5.336 | -0.178 |
|  | Gh_A11G2579.1 | GH3.17 | Indole-3-acetic acid-amido synthetase GH3.17 | A11 | 86,008,662 | 86,012,734 | - | 4,073 | 2,460 | 2,460 | 40.6 | 7 | 351.4 | 268.8 | 819 | 91.956 | -11.5 | 5.023 | -0.111 |
|  | Gh_A11G3061.1 | GH3.5 | Probable indole-3-acetic acid-amido synthetase GH3.5 | A11 | 110 | 3,248 | + | 3,139 | 1,758 | 1,758 | 41.2 | 4 | 439.5 | 460.3 | 585 | 65.707 | -10.5 | 5.085 | -0.114 |
|  | Gh_A11G3064.1 | GH3.5 | Probable indole-3-acetic acid-amido synthetase GH3.5 | A11 | 12,025 | 15,303 | + | 3,279 | 1,758 | 1,758 | 41.3 | 4 | 439.5 | 507 | 585 | 65.849 | -9 | 5.322 | -0.137 |
|  | Gh_A12G0181.1 | GH3.1 | Probable indole-3-acetic acid-amido synthetase GH3.1 | A12 | 2,676,124 | 2,678,101 | - | 1,978 | 1,794 | 1,794 | 48 | 3 | 598 | 92 | 597 | 67.886 | -1 | 6.386 | -0.322 |
|  | Gh_A13G0392.1 | GH3.1 | Probable indole-3-acetic acid-amido synthetase GH3.1 | A13 | 5,213,724 | 5,216,267 | + | 2,544 | 1,809 | 1,809 | 49.4 | 3 | 603 | 367.5 | 602 | 68.095 | -7.5 | 5.63 | -0.249 |
|  | Gh_A13G0480.1 | GH3.5 | Probable indole-3-acetic acid-amido synthetase GH3.5 | A13 | 10,393,456 | 10,395,665 | + | 2,210 | 1,797 | 1,797 | 45.1 | 4 | 449.3 | 137.7 | 598 | 67.232 | -7 | 5.629 | -0.139 |
|  | Gh_D01G0162.1 | GH3.5 | Probable indole-3-acetic acid-amido synthetase GH3.5 | D01 | 1,212,787 | 1,215,546 | + | 2,760 | 1,797 | 1,797 | 43.9 | 4 | 449.3 | 321 | 598 | 68.242 | 2 | 6.739 | -0.256 |
|  | Gh_D01G0557.1 | GH3.6 | Indole-3-acetic acid-amido synthetase GH3.6 | D01 | 7,266,590 | 7,268,663 | + | 2,074 | 1,842 | 1,842 | 43.7 | 3 | 614 | 116 | 613 | 69.477 | -2.5 | 6.157 | -0.26 |
|  | Gh_D01G0559.1 | GH3.6 | Indole-3-acetic acid-amido synthetase GH3.6 | D01 | 7,427,672 | 7,429,831 | + | 2,160 | 1,842 | 1,842 | 43.5 | 3 | 614 | 159 | 613 | 69.183 | -0.5 | 6.429 | -0.286 |
|  | Gh_D02G1794.1 | GH3.17 | Indole-3-acetic acid-amido synthetase GH3.17 | D02 | 60,662,189 | 60,664,648 | - | 2,460 | 1,935 | 1,935 | 42.2 | 5 | 387 | 131.3 | 644 | 73.258 | 4 | 7.15 | -0.31 |
|  | Gh_D02G2045.1 | GH3.1 | Probable indole-3-acetic acid-amido synthetase GH3.1 | D02 | 64,687,129 | 64,689,111 | - | 1,983 | 1,797 | 1,797 | 49 | 3 | 599 | 93 | 598 | 67.883 | 3 | 7.017 | -0.331 |
|  | Gh_D03G0465.1 | GH3.17 | Indole-3-acetic acid-amido synthetase GH3.17 | D03 | 7,344,056 | 7,347,201 | - | 3,146 | 1,758 | 1,758 | 40.8 | 4 | 439.5 | 462.7 | 585 | 66.064 | -3.5 | 5.817 | -0.218 |
|  | Gh_D04G0260.1 | GH3.17 | Indole-3-acetic acid-amido synthetase GH3.17 | D04 | 3,849,711 | 3,884,644 | + | 34,934 | 1,578 | 1,578 | 41.1 | 6 | 263 | 6,671.20 | 525 | 59.295 | -7 | 5.194 | -0.169 |
|  | Gh_D04G0261.1 | GH3.17 | Indole-3-acetic acid-amido synthetase GH3.17 | D04 | 3,888,030 | 3,891,848 | + | 3,819 | 1,695 | 1,695 | 41.1 | 5 | 339 | 531 | 564 | 63.494 | -5.5 | 5.47 | -0.213 |
|  | Gh_D04G0262.1 | GH3.17 | Indole-3-acetic acid-amido synthetase GH3.17 | D04 | 3,896,109 | 3,900,571 | + | 4,463 | 1,758 | 1,758 | 41.4 | 4 | 439.5 | 901.7 | 585 | 66.098 | -5.5 | 5.48 | -0.206 |
|  | Gh_D04G1375.1 | GH3.17 | Indole-3-acetic acid-amido synthetase GH3.17 | D04 | 44,607,387 | 44,608,227 | - | 841 | 729 | 729 | 39.9 | 2 | 364.5 | 112 | 242 | 27.77 | 1 | 6.913 | -0.141 |
|  | Gh_D05G3386.1 | GH3.17 | Indole-3-acetic acid-amido synthetase GH3.17 | D05 | 54,980,347 | 54,983,447 | - | 3,101 | 1,431 | 1,431 | 41 | 6 | 238.5 | 334 | 476 | 53.754 | -8 | 5.085 | -0.3 |
|  | Gh_D05G3387.1 | GH3.17 | Indole-3-acetic acid-amido synthetase GH3.17 | D05 | 54,990,165 | 54,994,031 | - | 3,867 | 1,785 | 1,785 | 41.8 | 4 | 446.3 | 694 | 594 | 67.877 | -3.5 | 5.935 | -0.235 |
|  | Gh_D07G1392.1 | GH3.5 | Probable indole-3-acetic acid-amido synthetase GH3.5 | D07 | 22,651,121 | 22,654,579 | - | 3,459 | 1,758 | 1,758 | 44 | 4 | 439.5 | 567 | 585 | 66.219 | -1 | 6.388 | -0.206 |
|  | Gh_D08G0262.1 | GH3.17 | Indole-3-acetic acid-amido synthetase GH3.17 | D08 | 2,507,000 | 2,519,666 | - | 12,667 | 3,414 | 3,414 | 41.1 | 9 | 379.3 | 1,156.60 | 1,137 | 126.749 | -1 | 6.424 | -0.198 |
|  | Gh_D08G1403.1 | GH3.5 | Probable indole-3-acetic acid-amido synthetase GH3.5 | D08 | 46,349,331 | 46,351,508 | - | 2,178 | 1,716 | 1,716 | 43.8 | 4 | 429 | 154 | 571 | 64.292 | -4.5 | 5.855 | -0.162 |
|  | Gh_D08G1987.1 | GH3.6 | Probable indole-3-acetic acid-amido synthetase GH3.6 | D08 | 58,324,153 | 58,326,094 | - | 1,942 | 1,749 | 1,749 | 42 | 3 | 583 | 96.5 | 582 | 65.501 | 0.5 | 6.569 | -0.146 |
|  | Gh_D11G0514.1 | GH3.1 | Probable indole-3-acetic acid-amido synthetase GH3.1 | D11 | 4,476,391 | 4,478,353 | + | 1,963 | 1,779 | 1,779 | 45.5 | 3 | 593 | 92 | 592 | 67.37 | -3 | 6.054 | -0.214 |
|  | Gh_D11G1005.1 | GH3.5 | Probable indole-3-acetic acid-amido synthetase GH3.5 | D11 | 8,813,399 | 8,816,393 | + | 2,995 | 1,758 | 1,758 | 41.9 | 4 | 439.5 | 412.3 | 585 | 65.793 | -6.5 | 5.671 | -0.143 |
|  | Gh_D11G1006.1 | GH3.5 | Probable indole-3-acetic acid-amido synthetase GH3.5 | D11 | 8,824,243 | 8,827,375 | + | 3,133 | 1,758 | 1,758 | 40.9 | 4 | 439.5 | 458.3 | 585 | 65.843 | -8.5 | 5.237 | -0.134 |
|  | Gh_D11G1209.1 | GH3.9 | Putative indole-3-acetic acid-amido synthetase GH3.9 | D11 | 11,289,480 | 11,292,643 | + | 3,164 | 1,788 | 1,788 | 44.5 | 4 | 447 | 458.7 | 595 | 66.824 | -8 | 5.29 | -0.147 |
|  | Gh_D11G1989.1 | GH3.1 | Probable indole-3-acetic acid-amido synthetase GH3.1 | D11 | 26,018,863 | 26,020,882 | + | 2,020 | 1,821 | 1,821 | 46.7 | 3 | 607 | 99.5 | 606 | 68.493 | -6.5 | 5.449 | -0.2 |
|  | Gh_D11G2943.1 | GH3.17 | Indole-3-acetic acid-amido synthetase GH3.17 | D11 | 60,015,511 | 60,017,611 | + | 2,101 | 1,755 | 1,755 | 40.3 | 4 | 438.8 | 115.3 | 584 | 66.084 | -8.5 | 4.997 | -0.133 |
|  | Gh_D11G2944.1 | GH3.17 | Indole-3-acetic acid-amido synthetase GH3.17 | D11 | 60,034,501 | 60,036,632 | + | 2,132 | 1,806 | 1,806 | 40 | 4 | 451.5 | 108.7 | 601 | 67.423 | -6.5 | 5.165 | -0.096 |
|  | Gh_D11G2945.1 | GH3.17 | Indole-3-acetic acid-amido synthetase GH3.17 | D11 | 60,052,389 | 60,054,363 | - | 1,975 | 1,827 | 1,827 | 39.5 | 2 | 913.5 | 148 | 608 | 68.552 | 5.5 | 8.027 | -0.199 |
|  | Gh_D12G0182.1 | GH3.1 | Probable indole-3-acetic acid-amido synthetase GH3.1 | D12 | 2,358,751 | 2,360,733 | - | 1,983 | 1,797 | 1,797 | 48 | 3 | 599 | 93 | 598 | 67.901 | -1 | 6.385 | -0.31 |
|  | Gh_D13G0434.1 | GH3.1 | Probable indole-3-acetic acid-amido synthetase GH3.1 | D13 | 4,919,812 | 4,922,365 | + | 2,554 | 1,809 | 1,809 | 49.4 | 3 | 603 | 372.5 | 602 | 68.037 | -7 | 5.741 | -0.242 |
|  | Gh_D13G0668.1 | GH3.5 | Probable indole-3-acetic acid-amido synthetase GH3.5 | D13 | 9,417,517 | 9,419,739 | - | 2,223 | 1,797 | 1,797 | 45.2 | 4 | 449.3 | 142 | 598 | 67.264 | -5.5 | 5.79 | -0.138 |
| **DD** | Gorai.001G156600.1 | GH3.5 | Probable indole-3-acetic acid-amido synthetase GH3.5 | Chr01 | 22,194,460 | 22,199,098 | - | 4,639 | 2,262 | 1,758 | 44 | 5 | 452.4 | 594.3 | 585 | 66.223 | -1 | 6.387 | -0.209 |
|  | Gorai.002G017500.1 | GH3.5 | Probable indole-3-acetic acid-amido synthetase GH3.5 | Chr02 | 1,171,712 | 1,175,758 | + | 4,047 | 2,021 | 1,803 | 44.2 | 4 | 505.3 | 675.3 | 600 | 68.419 | 4.5 | 7.027 | -0.274 |
|  | Gorai.002G079500.1 | GH3.6 | Indole-3-acetic acid-amido synthetase GH3.6 | Chr02 | 9,621,997 | 9,624,525 | + | 2,529 | 2,294 | 1,842 | 44 | 3 | 764.7 | 117.5 | 613 | 69.446 | -2 | 6.205 | -0.259 |
|  | Gorai.002G079600.1 | GH3.6 | Indole-3-acetic acid-amido synthetase GH3.6 | Chr02 | 9,774,365 | 9,777,022 | + | 2,658 | 2,340 | 1,842 | 43.4 | 3 | 780 | 159 | 613 | 69.196 | -1.5 | 6.263 | -0.29 |
|  | Gorai.002G107400.1 | GH3.17 | Indole-3-acetic acid-amido synthetase GH3.17 | Chr02 | 14,510,202 | 14,512,878 | - | 2,677 | 2,057 | 1,617 | 39.8 | 4 | 514.3 | 206.7 | 538 | 60.319 | 2 | 6.884 | -0.127 |
|  | Gorai.003G050400.1 | GH3.17 | Indole-3-acetic acid-amido synthetase GH3.17 | Chr03 | 7,294,213 | 7,297,704 | - | 3,492 | 2,100 | 1,743 | 40.8 | 4 | 525 | 464 | 580 | 65.51 | -4 | 5.783 | -0.206 |
|  | Gorai.004G029600.1 | GH3.17 | Indole-3-acetic acid-amido synthetase GH3.17 | Chr04 | 2,343,637 | 2,352,263 | - | 8,627 | 3,603 | 3,414 | 41.2 | 8 | 450.4 | 717.7 | 1,137 | 126.531 | -5 | 6.047 | -0.203 |
|  | Gorai.004G152800.1 | GH3.5 | Probable indole-3-acetic acid-amido synthetase GH3.5 | Chr04 | 43,442,543 | 43,446,211 | - | 3,669 | 2,116 | 1,716 | 43.8 | 5 | 423.2 | 388.3 | 571 | 64.302 | -4.5 | 5.855 | -0.16 |
|  | Gorai.004G214900.1 | GH3.6 | Probable indole-3-acetic acid-amido synthetase GH3.6 | Chr04 | 54,776,104 | 54,778,499 | - | 2,396 | 2,201 | 1,749 | 42.1 | 3 | 733.7 | 97.5 | 582 | 65.455 | 0 | 6.51 | -0.151 |
|  | Gorai.005G197700.1 | GH3.17 | Indole-3-acetic acid-amido synthetase GH3.17 | Chr05 | 57,193,347 | 57,196,166 | - | 2,820 | 2,295 | 1,935 | 42.1 | 5 | 459 | 131.3 | 644 | 73.256 | 5 | 7.331 | -0.298 |
|  | Gorai.005G208000.1 | GH3.6 | Indole-3-acetic acid-amido synthetase GH3.6 | Chr05 | 58,844,803 | 58,847,154 | + | 2,352 | 2,148 | 1,848 | 43.2 | 3 | 716 | 102 | 615 | 69.169 | -7.5 | 5.07 | -0.181 |
|  | Gorai.005G234200.1 | GH3.1 | Probable indole-3-acetic acid-amido synthetase GH3.1 | Chr05 | 61,624,195 | 61,626,605 | - | 2,411 | 2,221 | 1,797 | 48.9 | 3 | 740.3 | 95 | 598 | 67.855 | 2.5 | 6.963 | -0.325 |
|  | Gorai.007G055900.1 | GH3.1 | Probable indole-3-acetic acid-amido synthetase GH3.1 | Chr07 | 3,949,918 | 3,952,344 | + | 2,427 | 2,249 | 1,779 | 45.6 | 3 | 749.7 | 89 | 592 | 67.374 | -6 | 5.572 | -0.196 |
|  | Gorai.007G106500.1 | GH3.5 | Probable indole-3-acetic acid-amido synthetase GH3.5 | Chr07 | 8,002,951 | 8,006,032 | + | 3,082 | 1,782 | 1,758 | 41.9 | 4 | 445.5 | 433.3 | 585 | 65.786 | -10.5 | 5.229 | -0.149 |
|  | Gorai.007G106600.1 | GH3.5 | Probable indole-3-acetic acid-amido synthetase GH3.5 | Chr07 | 8,019,174 | 8,023,041 | + | 3,868 | 1,950 | 1,758 | 41 | 5 | 390 | 479.5 | 585 | 65.838 | -9.5 | 5.157 | -0.131 |
|  | Gorai.007G129200.1 | GH3.9 | Putative indole-3-acetic acid-amido synthetase GH3.9 | Chr07 | 10,363,505 | 10,367,041 | + | 3,537 | 2,165 | 1,788 | 44.3 | 4 | 541.3 | 457.3 | 595 | 66.938 | -8.5 | 5.196 | -0.151 |
|  | Gorai.007G219500.1 | GH3.1 | Probable indole-3-acetic acid-amido synthetase GH3.1 | Chr07 | 25,218,502 | 25,220,988 | + | 2,487 | 2,288 | 1,821 | 47 | 3 | 762.7 | 99.5 | 606 | 68.479 | -6.5 | 5.449 | -0.2 |
|  | Gorai.007G323400.1 | GH3.17 | Indole-3-acetic acid-amido synthetase GH3.17 | Chr07 | 54,137,383 | 54,140,068 | + | 2,686 | 1,943 | 1,824 | 40.1 | 5 | 388.6 | 185.8 | 607 | 68.851 | -8.5 | 4.997 | -0.051 |
|  | Gorai.007G323500.1 | GH3.17 | Indole-3-acetic acid-amido synthetase GH3.17 | Chr07 | 54,157,092 | 54,159,325 | + | 2,234 | 1,908 | 1,806 | 40 | 4 | 477 | 108.7 | 601 | 67.395 | -7.5 | 5.083 | -0.094 |
|  | Gorai.007G323600.1 | GH3.17 | Indole-3-acetic acid-amido synthetase GH3.17 | Chr07 | 54,174,873 | 54,176,913 | - | 2,041 | 1,821 | 1,755 | 39.4 | 3 | 607 | 110 | 584 | 66.036 | 2 | 7.453 | -0.18 |
|  | Gorai.008G021400.1 | GH3.1 | Probable indole-3-acetic acid-amido synthetase GH3.1 | Chr08 | 2,430,283 | 2,432,737 | - | 2,455 | 2,268 | 1,797 | 48 | 3 | 756 | 93.5 | 598 | 67.865 | -1.5 | 6.307 | -0.297 |
|  | Gorai.009G381700.1 | GH3.17 | Indole-3-acetic acid-amido synthetase GH3.17 | Chr09 | 51,813,196 | 51,814,560 | - | 1,365 | 1,182 | 1,182 | 40.9 | 4 | 295.5 | 61 | 393 | 44.513 | 0 | 6.506 | -0.095 |
|  | Gorai.009G382000.1 | GH3.17 | Indole-3-acetic acid-amido synthetase GH3.17 | Chr09 | 51,842,003 | 51,844,850 | - | 2,848 | 2,094 | 1,614 | 41.6 | 4 | 523.5 | 251.3 | 537 | 60.111 | 0 | 6.518 | -0.171 |
|  | Gorai.009G382100.1 | GH3.17 | Indole-3-acetic acid-amido synthetase GH3.17 | Chr09 | 51,857,544 | 51,858,101 | - | 558 | 558 | 558 | 43.5 | 1 | 558 | No intron | 185 | 21.263 | 3 | 8.304 | -0.151 |
|  | Gorai.009G382300.1 | GH3.5 | Indole-3-acetic acid-amido synthetase GH3.5 | Chr09 | 51,898,282 | 51,898,816 | - | 535 | 535 | 312 | 39.4 | 1 | 535 | No intron | 103 | 11.903 | -5 | 4.558 | -0.478 |
|  | Gorai.009G382400.1 | GH3.17 | Indole-3-acetic acid-amido synthetase GH3.17 | Chr09 | 51,902,576 | 51,904,555 | - | 1,980 | 930 | 930 | 41.4 | 3 | 310 | 525 | 309 | 35.566 | -3 | 5.524 | -0.345 |
|  | Gorai.009G382900.1 | GH3.17 | Indole-3-acetic acid-amido synthetase GH3.17 | Chr09 | 51,960,247 | 51,963,002 | - | 2,756 | 1,674 | 1,674 | 41 | 5 | 334.8 | 202.5 | 557 | 62.227 | -10 | 5.026 | -0.162 |
|  | Gorai.009G383300.1 | GH3.17 | Indole-3-acetic acid-amido synthetase GH3.17 | Chr09 | 52,020,667 | 52,031,450 | - | 10,784 | 1,518 | 1,518 | 40.8 | 6 | 253 | 1,847.60 | 505 | 56.765 | -7.5 | 5.251 | -0.194 |
|  | Gorai.009G383400.1 | GH3.17 | Indole-3-acetic acid-amido synthetase GH3.17 | Chr09 | 52,034,041 | 52,038,037 | - | 3,997 | 1,977 | 1,785 | 41.9 | 4 | 494.3 | 673.3 | 594 | 67.914 | -3.5 | 5.935 | -0.239 |
|  | Gorai.009G387200.1 | GH3.17 | Indole-3-acetic acid-amido synthetase GH3.17 | Chr09 | 52,488,764 | 52,489,721 | - | 958 | 879 | 702 | 41.2 | 2 | 439.5 | 79 | 233 | 26.854 | -1.5 | 5.823 | -0.322 |
|  | Gorai.010G178700.1 | GH3.5 | Probable indole-3-acetic acid-amido synthetase GH3.5 | Chr10 | 52,458,219 | 52,458,639 | + | 421 | 421 | 207 | 38.2 | 1 | 421 | No intron | 68 | 8.024 | -5.5 | 4.322 | -0.063 |
|  | Gorai.012G029800.1 | GH3.17 | Indole-3-acetic acid-amido synthetase GH3.17 | Chr12 | 3,684,238 | 3,696,952 | - | 12,715 | 1,927 | 1,743 | 41.5 | 4 | 481.8 | 3,596.00 | 580 | 65.724 | -3 | 5.969 | -0.265 |
|  | Gorai.012G029900.1 | GH3.17 | Indole-3-acetic acid-amido synthetase GH3.17 | Chr12 | 3,699,804 | 3,702,174 | - | 2,371 | 1,773 | 1,773 | 40.9 | 5 | 354.6 | 149.5 | 590 | 66.58 | -10 | 5.029 | -0.193 |
|  | Gorai.012G030000.1 | GH3.17 | Indole-3-acetic acid-amido synthetase GH3.17 | Chr12 | 3,714,499 | 3,717,266 | - | 2,768 | 2,289 | 1,743 | 40.8 | 4 | 572.3 | 159.7 | 580 | 65.694 | -5 | 5.492 | -0.213 |
|  | Gorai.012G030200.1 | GH3.17 | Indole-3-acetic acid-amido synthetase GH3.17 | Chr12 | 3,727,775 | 3,730,173 | - | 2,399 | 2,056 | 1,743 | 40.7 | 4 | 514 | 114.3 | 580 | 65.625 | -2.5 | 6.098 | -0.202 |
|  | Gorai.012G126200.1 | GH3.17 | Indole-3-acetic acid-amido synthetase GH3.17 | Chr12 | 29,086,856 | 29,088,749 | - | 1,894 | 1,631 | 1,392 | 40.7 | 4 | 407.8 | 87.7 | 463 | 52.289 | 6.5 | 7.89 | -0.062 |
|  | Gorai.013G048400.1 | GH3.1 | Probable indole-3-acetic acid-amido synthetase GH3.1 | Chr13 | 4,308,939 | 4,311,834 | + | 2,896 | 2,153 | 1,809 | 49.9 | 3 | 717.7 | 371.5 | 602 | 67.995 | -7 | 5.741 | -0.267 |
|  | Gorai.013G074800.1 | GH3.5 | Probable indole-3-acetic acid-amido synthetase GH3.5 | Chr13 | 9,004,191 | 9,006,736 | - | 2,546 | 2,121 | 1,797 | 45.4 | 4 | 530.3 | 141.7 | 598 | 67.308 | -6.5 | 5.647 | -0.147 |
| **AA** | Ga01G0190.1 | GH3.10 | Indole-3-acetic acid-amido synthetase GH3.10 | Chr01 | 1,359,446 | 1,363,357 | + | 3,912 | 1,803 | 1,803 | 44.5 | 4 | 450.8 | 703 | 600 | 68.524 | 6 | 7.294 | -0.291 |
|  | Ga01G0651.1 | GH3.17 | Indole-3-acetic acid-amido synthetase GH3.17 | Chr01 | 8,653,805 | 8,657,015 | + | 3,211 | 1,554 | 1,554 | 41.9 | 5 | 310.8 | 414.3 | 517 | 57.988 | -2.5 | 5.968 | -0.039 |
|  | Ga01G0770.1 | GH3.6 | Indole-3-acetic acid-amido synthetase GH3.6 | Chr01 | 10,959,126 | 10,961,202 | + | 2,077 | 1,842 | 1,842 | 43.9 | 3 | 614 | 117.5 | 613 | 69.484 | -1.5 | 6.301 | -0.282 |
|  | Ga01G0771.1 | GH3.6 | Indole-3-acetic acid-amido synthetase GH3.6 | Chr01 | 11,115,630 | 11,117,791 | + | 2,162 | 1,842 | 1,842 | 43.6 | 3 | 614 | 160 | 613 | 69.241 | -0.5 | 6.43 | -0.29 |
|  | Ga01G1048.1 | GH3.17 | Indole-3-acetic acid-amido synthetase GH3.17 | Chr01 | 18,287,522 | 18,292,798 | - | 5,277 | 1,386 | 1,386 | 38.8 | 8 | 173.3 | 555.9 | 461 | 51.708 | 12 | 8.727 | -0.094 |
|  | Ga02G0485.1 | GH3.17 | Indole-3-acetic acid-amido synthetase GH3.17 | Chr02 | 8,121,902 | 8,126,449 | + | 4,548 | 1,761 | 1,761 | 40.1 | 5 | 352.2 | 696.8 | 586 | 66.342 | 0 | 6.514 | -0.199 |
|  | Ga02G0486.1 | GH3.17 | Indole-3-acetic acid-amido synthetase GH3.17 | Chr02 | 8,131,486 | 8,133,938 | + | 2,453 | 1,677 | 1,677 | 40 | 5 | 335.4 | 194 | 558 | 63.113 | 0.5 | 6.607 | -0.21 |
|  | Ga03G2055.1 | GH3.17 | Indole-3-acetic acid-amido synthetase GH3.17 | Chr03 | 127,054,502 | 127,056,953 | - | 2,452 | 1,935 | 1,935 | 42.5 | 5 | 387 | 129.3 | 644 | 73.195 | 0 | 6.511 | -0.281 |
|  | Ga03G2153.1 | GH3.6 | Indole-3-acetic acid-amido synthetase GH3.6 | Chr03 | 128,887,675 | 128,889,723 | + | 2,049 | 1,848 | 1,848 | 43.3 | 3 | 616 | 100.5 | 615 | 69.035 | -5.5 | 5.339 | -0.165 |
|  | Ga03G2421.1 | GH3.1 | Probable indole-3-acetic acid-amido synthetase GH3.1 | Chr03 | 132,743,060 | 132,745,054 | - | 1,995 | 1,797 | 1,797 | 49 | 3 | 599 | 99 | 598 | 67.776 | 2 | 6.832 | -0.314 |
|  | Ga04G0493.1 | GH3.17 | Indole-3-acetic acid-amido synthetase GH3.17 | Chr04 | 7,332,481 | 7,334,521 | + | 2,041 | 1,617 | 1,617 | 41.1 | 5 | 323.4 | 106 | 538 | 60.688 | -2.5 | 6.087 | -0.121 |
|  | Ga04G1833.1 | GH3.17 | Indole-3-acetic acid-amido synthetase GH3.17 | Chr04 | 94,125,836 | 94,127,910 | + | 2,075 | 1,731 | 1,731 | 40.8 | 4 | 432.8 | 114.7 | 576 | 65.016 | -7.5 | 5.251 | -0.201 |
|  | Ga04G1834.1 | GH3.17 | Indole-3-acetic acid-amido synthetase GH3.17 | Chr04 | 94,144,369 | 94,148,320 | + | 3,952 | 1,770 | 1,770 | 39.9 | 5 | 354 | 545.5 | 589 | 66.617 | -5 | 5.494 | -0.195 |
|  | Ga04G1835.1 | GH3.17 | Indole-3-acetic acid-amido synthetase GH3.17 | Chr04 | 94,156,976 | 94,159,332 | + | 2,357 | 1,722 | 1,722 | 40.5 | 6 | 287 | 127 | 573 | 64.729 | -4.5 | 5.636 | -0.236 |
|  | Ga04G1836.1 | GH3.17 | Indole-3-acetic acid-amido synthetase GH3.17 | Chr04 | 94,162,286 | 94,166,226 | + | 3,941 | 1,752 | 1,752 | 41 | 5 | 350.4 | 547.3 | 583 | 65.772 | -4.5 | 5.63 | -0.203 |
|  | Ga04G1837.1 | GH3.17 | Indole-3-acetic acid-amido synthetase GH3.17 | Chr04 | 94,222,526 | 94,228,085 | + | 5,560 | 1,731 | 1,731 | 40.8 | 4 | 432.8 | 1,276.30 | 576 | 65.266 | -3.5 | 5.813 | -0.224 |
|  | Ga05G3894.1 | GH3.17 | Indole-3-acetic acid-amido synthetase GH3.17 | Chr05 | 89,656,389 | 89,658,929 | - | 2,541 | 1,803 | 1,803 | 40.7 | 4 | 450.8 | 246 | 600 | 67.502 | -10.5 | 5.067 | -0.215 |
|  | Ga05G3895.1 | GH3.17 | Indole-3-acetic acid-amido synthetase GH3.17 | Chr05 | 89,668,112 | 89,672,155 | - | 4,044 | 1,734 | 1,734 | 41.9 | 5 | 346.8 | 577.5 | 577 | 66.002 | -6 | 5.474 | -0.256 |
|  | Ga07G1535.1 | GH3.5 | Jasmonic acid-amido synthetase JAR1 | Chr07 | 29,908,628 | 29,913,371 | - | 4,744 | 1,821 | 1,821 | 44.2 | 5 | 364.2 | 730.8 | 606 | 68.453 | 1.5 | 6.671 | -0.18 |
|  | Ga08G0305.1 | GH3.17 | Indole-3-acetic acid-amido synthetase GH3.17 | Chr08 | 2,822,845 | 2,831,248 | - | 8,404 | 3,351 | 3,351 | 40.9 | 8 | 418.9 | 721.9 | 1,116 | 124.403 | -1 | 6.427 | -0.148 |
|  | Ga08G1506.1 | GH3.5 | Jasmonic acid-amido synthetase JAR1 | Chr08 | 102,449,999 | 102,452,163 | - | 2,165 | 1,716 | 1,716 | 43.2 | 4 | 429 | 149.7 | 571 | 64.264 | -3 | 6.049 | -0.159 |
|  | Ga08G2175.1 | GH3.6 | Probable indole-3-acetic acid-amido synthetase GH3.6 | Chr08 | 120,349,492 | 120,351,438 | - | 1,947 | 1,749 | 1,749 | 41.7 | 3 | 583 | 99 | 582 | 65.391 | 1.5 | 6.691 | -0.11 |
|  | Ga11G0584.1 | GH3.17 | Indole-3-acetic acid-amido synthetase GH3.17 | Chr11 | 8,978,853 | 8,981,047 | + | 2,195 | 1,776 | 1,776 | 40.5 | 4 | 444 | 139.7 | 591 | 66.149 | -8.5 | 4.987 | -0.092 |
|  | Ga11G0586.1 | GH3.17 | Indole-3-acetic acid-amido synthetase GH3.17 | Chr11 | 9,007,764 | 9,009,877 | + | 2,114 | 1,692 | 1,692 | 39.8 | 4 | 423 | 140.7 | 563 | 63.584 | -3.5 | 5.788 | -0.169 |
|  | Ga11G0589.1 | GH3.17 | Indole-3-acetic acid-amido synthetase GH3.17 | Chr11 | 9,039,681 | 9,041,876 | + | 2,196 | 1,686 | 1,686 | 40.5 | 6 | 281 | 102 | 561 | 63.116 | -7 | 5.195 | -0.16 |
|  | Ga11G0590.1 | GH3.17 | Indole-3-acetic acid-amido synthetase GH3.17 | Chr11 | 9,047,062 | 9,048,952 | + | 1,891 | 1,512 | 1,512 | 39.9 | 5 | 302.4 | 94.8 | 503 | 56.578 | -2.5 | 5.825 | -0.172 |
|  | Ga11G0592.1 | GH3.17 | Indole-3-acetic acid-amido synthetase GH3.17 | Chr11 | 9,124,949 | 9,127,088 | - | 2,140 | 1,806 | 1,806 | 39.9 | 4 | 451.5 | 111.3 | 601 | 67.553 | -5.5 | 5.255 | -0.105 |
|  | Ga11G0593.1 | GH3.17 | Indole-3-acetic acid-amido synthetase GH3.17 | Chr11 | 9,153,091 | 9,155,646 | - | 2,556 | 1,818 | 1,818 | 40.2 | 5 | 363.6 | 184.5 | 605 | 68.549 | -4 | 5.5 | -0.07 |
|  | Ga11G1865.1 | GH3.1 | Probable indole-3-acetic acid-amido synthetase GH3.1 | Chr11 | 86,353,616 | 86,355,635 | - | 2,020 | 1,821 | 1,821 | 47 | 3 | 607 | 99.5 | 606 | 68.491 | -6 | 5.458 | -0.216 |
|  | Ga11G2835.1 | GH3.9 | Putative indole-3-acetic acid-amido synthetase GH3.9 | Chr11 | 111,834,370 | 111,837,538 | - | 3,169 | 1,788 | 1,788 | 43.9 | 4 | 447 | 460.3 | 595 | 67.017 | -5.5 | 5.565 | -0.126 |
|  | Ga11G3055.1 | GH3.5 | Jasmonic acid-amido synthetase JAR1 | Chr11 | 115,165,800 | 115,168,939 | - | 3,140 | 1,758 | 1,758 | 41.1 | 4 | 439.5 | 460.7 | 585 | 65.725 | -8.5 | 5.241 | -0.113 |
|  | Ga11G3056.1 | GH3.5 | Jasmonic acid-amido synthetase JAR1 | Chr11 | 115,172,454 | 115,175,690 | - | 3,237 | 1,758 | 1,758 | 41.2 | 4 | 439.5 | 493 | 585 | 65.875 | -8 | 5.426 | -0.123 |
|  | Ga11G3574.1 | GH3.1 | Probable indole-3-acetic acid-amido synthetase GH3.1 | Chr11 | 119,853,259 | 119,855,219 | - | 1,961 | 1,779 | 1,779 | 45.5 | 3 | 593 | 91 | 592 | 67.455 | -3 | 6.052 | -0.214 |
|  | Ga13G0436.1 | GH3.1 | Probable indole-3-acetic acid-amido synthetase GH3.1 | Chr13 | 5,098,060 | 5,100,605 | + | 2,546 | 1,809 | 1,809 | 49.6 | 3 | 603 | 368.5 | 602 | 68.04 | -9.5 | 5.393 | -0.258 |
|  | Ga13G0765.1 | GH3.10 | Indole-3-acetic acid-amido synthetase GH3.10 | Chr13 | 12,348,472 | 12,350,681 | - | 2,210 | 1,797 | 1,797 | 45.2 | 4 | 449.3 | 137.7 | 598 | 67.235 | -6 | 5.768 | -0.155 |
|  | Ga14G0127.1 | GH3.1 | Probable indole-3-acetic acid-amido synthetase GH3.1 | tig00000498 | 199,773 | 201,750 | - | 1,978 | 1,794 | 1,794 | 48 | 3 | 598 | 92 | 597 | 67.973 | 0.5 | 6.587 | -0.322 |
