# Supplementary material for: Functional characterization of Gh_A08G1120 (GH3.5) gene reveal their significant role in enhancing drought and salt stress tolerance in cotton
Source: BMC Genet. 2019 Jul 23;20:62. doi: 10.1186/s12863-019-0756-6 (PMC6651995; doi:10.1186/s12863-019-0756-6)
Supplement: Supplementary file 3 — Table S3. Go analysis of the upland cotton, G. hirsutum GH3 genes. (DOCX 21 kb) [file 12863_2019_756_MOESM3_ESM.docx]

Table S3: GO analysis of the upland cotton, *G. hirsutum GH3* genes

| Gh_D11G1005 | GH3.5 | Probable indole-3-acetic acid-amido synthetase GH3.5 | 4 | P:GO:0009694 | P:jasmonic acid metabolic process |
| --- | --- | --- | --- | --- | --- |
|  |  |  |  | F:GO:0080123 | F:jasmonate-amino synthetase activity |
|  |  |  |  | P:GO:0009611 | P:response to wounding |
|  |  |  |  | P:GO:0009864 | P:induced systemic resistance, jasmonic acid mediated signaling pathway |
| Gh_D11G1006 | GH3.5 | Probable indole-3-acetic acid-amido synthetase GH3.5 | 4 | P:GO:0009694 | P:jasmonic acid metabolic process |
|  |  |  |  | F:GO:0080123 | F:jasmonate-amino synthetase activity |
|  |  |  |  | P:GO:0009611 | P:response to wounding |
|  |  |  |  | P:GO:0009864 | P:induced systemic resistance, jasmonic acid mediated signaling pathway |
| Gh_A01G2047 | GH3.5 | Probable indole-3-acetic acid-amido synthetase GH3.5 | 1 | C:GO:0009507 | C:chloroplast |
| Gh_A07G1280 | GH3.5 | Probable indole-3-acetic acid-amido synthetase GH3.5 | 4 | P:GO:0009694 | P:jasmonic acid metabolic process |
|  |  |  |  | F:GO:0080123 | F:jasmonate-amino synthetase activity |
|  |  |  |  | P:GO:0009611 | P:response to wounding |
|  |  |  |  | P:GO:0009864 | P:induced systemic resistance, jasmonic acid mediated signaling pathway |
| Gh_A08G1120 | GH3.5 | Probable indole-3-acetic acid-amido synthetase GH3.5 | 4 | P:GO:0009694 | P:jasmonic acid metabolic process |
|  |  |  |  | F:GO:0080123 | F:jasmonate-amino synthetase activity |
|  |  |  |  | P:GO:0009611 | P:response to wounding |
|  |  |  |  | P:GO:0009864 | P:induced systemic resistance, jasmonic acid mediated signaling pathway |
| Gh_D11G1209 | GH3.9 | Putative indole-3-acetic acid-amido synthetase GH3.9 | 1 | C:GO:0009941 | C:chloroplast envelope |
| Gh_D07G1392 | GH3.5 | Probable indole-3-acetic acid-amido synthetase GH3.5 | 4 | P:GO:0009694 | P:jasmonic acid metabolic process |
|  |  |  |  | F:GO:0080123 | F:jasmonate-amino synthetase activity |
|  |  |  |  | P:GO:0009611 | P:response to wounding |
|  |  |  |  | P:GO:0009864 | P:induced systemic resistance, jasmonic acid mediated signaling pathway |
| Gh_A13G0480 | GH3.5 | Probable indole-3-acetic acid-amido synthetase GH3.5 | 1 | C:GO:0009507 | C:chloroplast |
| Gh_A11G1054 | GH3.9 | Putative indole-3-acetic acid-amido synthetase GH3.9 | 1 | C:GO:0009941 | C:chloroplast envelope |
| Gh_D01G0162 | GH3.5 | Probable indole-3-acetic acid-amido synthetase GH3.5 | 1 | C:GO:0009507 | C:chloroplast |
| Gh_D08G1403 | GH3.5 | Probable indole-3-acetic acid-amido synthetase GH3.5 | 4 | P:GO:0009694 | P:jasmonic acid metabolic process |
|  |  |  |  | F:GO:0080123 | F:jasmonate-amino synthetase activity |
|  |  |  |  | P:GO:0009611 | P:response to wounding |
|  |  |  |  | P:GO:0009864 | P:induced systemic resistance, jasmonic acid mediated signaling pathway |
| Gh_D13G0668 | GH3.5 | Probable indole-3-acetic acid-amido synthetase GH3.5 | 1 | C:GO:0009507 | C:chloroplast |
| Gh_A11G3064 | GH3.5 | Probable indole-3-acetic acid-amido synthetase GH3.5 | 4 | P:GO:0009694 | P:jasmonic acid metabolic process |
|  |  |  |  | F:GO:0080123 | F:jasmonate-amino synthetase activity |
|  |  |  |  | P:GO:0009611 | P:response to wounding |
|  |  |  |  | P:GO:0009864 | P:induced systemic resistance, jasmonic acid mediated signaling pathway |
| Gh_A11G3061 | GH3.5 | Probable indole-3-acetic acid-amido synthetase GH3.5 | 4 | P:GO:0009694 | P:jasmonic acid metabolic process |
|  |  |  |  | F:GO:0080123 | F:jasmonate-amino synthetase activity |
|  |  |  |  | P:GO:0009611 | P:response to wounding |
|  |  |  |  | P:GO:0009864 | P:induced systemic resistance, jasmonic acid mediated signaling pathway |
